# Biological activities of *Usnea lethariiformis* lichen extracts and UHPLC-ESI-QTOF-MS analysis of their secondary metabolites

**DOI:** 10.3389/fphar.2024.1508835

**Published:** 2025-01-06

**Authors:** Mauricio Piñeiro, Sofía Manrique, Jessica Gómez, Juan Manuel Rodriguez, Patricia Barrera, Duilio Caballero, Miguel A. Sosa, Gabriel Vargas-Arana, Alejandro Tapia, Beatriz Lima, Mario J. Simirgiotis

**Affiliations:** ^1^ Instituto de Biotecnología-Instituto de Ciencias Básicas, Universidad Nacional de San Juan (UNSJ), San Juan, Argentina; ^2^ Consejo Nacional de Investigaciones Científicas y Técnicas (CONICET), CABA, Buenos Aires, Argentina; ^3^ Instituto de Investigaciones Biológicas y Tecnológicas, Centro de Ecología y Recursos Naturales Renovables, Facultad de Ciencias Exactas, Físicas y Naturales (CONICET – Universidad Nacional de Córdoba), Córdoba, Argentina; ^4^ Instituto de Histología y Embriología “Dr. Mario H. Burgos”, Facultad de Ciencias Médicas, Universidad Nacional de Cuyo-CONICET, Mendoza, Argentina; ^5^ Laboratorio Hospital Marcial Quiroga, San Juan, Argentina; ^6^ Laboratorio de Química de Productos Naturales, Instituto de Investigaciones de la Amazonía Peruana, Iquitos, Peru; ^7^ Facultad de Industrias Alimentarias, Universidad Nacional de la Amazonía Peruana, Iquitos, Peru; ^8^ Instituto de Farmacia, Facultad de Ciencias, Universidad Austral de Chile, Valdivia, Chile

**Keywords:** trypanocidal, antibacterial, antifungal, nematicidal, *Trypanosoma cruzi*, *Meloidogyne incognita*

## Abstract

This research was designed to investigate the metabolite profiling, phenolics content, and the trypanocidal, nematicidal, antibacterial, antifungal, and free radical scavenging properties of *Usnea lethariiformis* Motyka. The air-dried *U. lethariiformis* material was extracted successively with dichloromethane and methanol (UlMeOH). Two phases were obtained from the extract with dichloromethane, one soluble in methanol (UlDCM-s) and the other insoluble (UlDCM-i). The metabolite profiling was obtained using ultra-high-resolution liquid chromatography coupled with electrospray ionization quadrupole time-of-flight mass spectrometry (UHPLC-ESI-QTOF-MS) system. The trypanocidal and nematicidal activities were determined according to standardized protocols. The antimicrobial activity was evaluated according to the Clinical and Laboratory Standards Institute (CLSI) rules. The total phenolic content of lichen extracts was determined by the Folin–Ciocalteu method. Antioxidant properties were assessed through 2,2-diphenyl-1-picrylhydrazyl (DPPH), Trolox equivalent antioxidant activity (TEAC), ferric-reducing antioxidant power (FRAP), and inhibition of lipid peroxidation in erythrocytes (ILP) assays, and the extracts exhibited robust antioxidant activity. Seventeen compounds were detected, of which thirteen were identified by UHPLC-ESI-QTOF-MS analysis, including depsides, depsidones, fatty acids, dibenzofurans, benzoic acids, and triterpenes. The UlDCM-s and UlMeOH extracts displayed strong trypanocidal activity against *Trypanosoma cruzi* epimastigotes at 50 μg/mL and 100 μg/mL and a nematicidal activity toward J2 *Meloidogyne incognita*, an important nematode infecting horticultural crops. Regarding the antimicrobial activity, the results showed that all bacteria and yeasts tested were inhibited by the different extracts with minimum inhibitory concentration (MIC) values between 25 μg/mL and 500 μg/mL. The UlDCM-s and UlMeOH extracts showed phenolic content of 107 mg and 48 mg gallic acid equivalents (GAE)/g dried extract, respectively. The UlDCM-s, UIDCM-i, and UlMeOH extracts showed moderate free radical scavenging activity in the DPPH, FRAP, and TEAC assays until 1 mg/mL and ILP tests at 250 μg/mL. The results indicated that *U. lethariiformis* may constitute a potential source of diverse bioactivities with application in the food, pharmaceutical, and agronomic industries.

## 1 Introduction

Diseases resistant to standard treatment modalities pose immense challenges to human and plant health worldwide. Beyond the considerable burden on quality of life, these issues strain healthcare infrastructures and economic resources (Tahami Monfared et al., 2022). Current estimates suggest that 80% of human infections are associated with pathogen biofilms, underscoring an urgent need for potent antibacterial agents (Kaczmarek, 2020). In the field of neglected diseases, Chagas disease (CD), caused by *Trypanosoma cruzi* and primarily transmitted by triatomine insects, is a major threat, especially in its chronic phase, where existing treatments show limited efficacy (Crespillo-Andújar et al., 2022).

**FIGURE 1:**
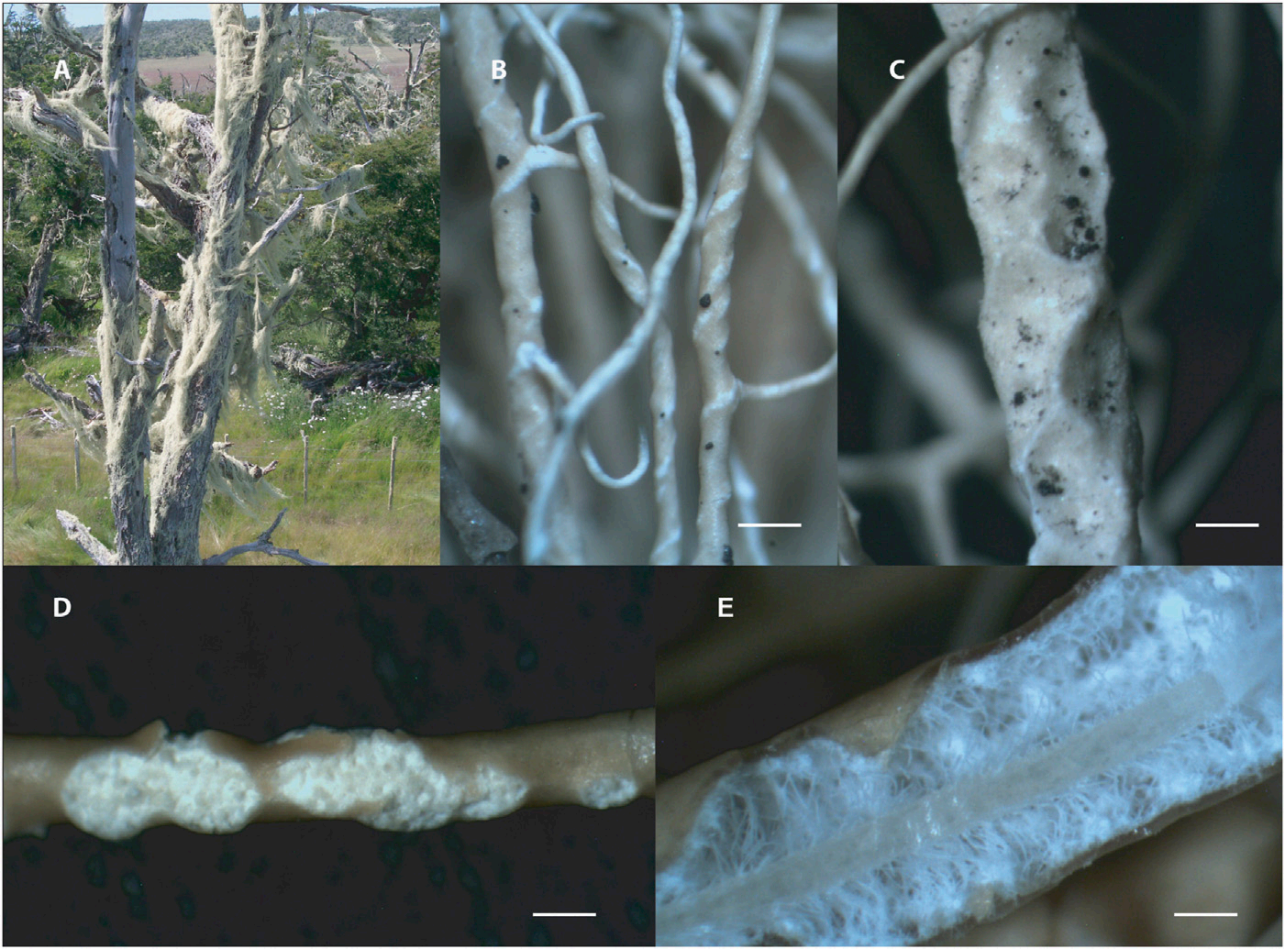
*Usnea lethariiformis*. **(A)** Tree completely covered by thalli of *U. lethariiformis*. **(B)** Terminal branches with twisted macula. **(C)** Branches with foveoles. **(D)** Large and confluent soralia with granular soredia. **(E)** Cortex, medulla, and axis in the longitudinal section. Scales: A = 3 mm, B, D, and E = 0.25 mm, C = 0.5 mm.

At the same time, the integrity of food safety is further compromised by plant-parasitic nematodes like *Meloidogyne incognita*, which undermine agricultural productivity by compromising root systems and nutrient uptake, leading to a global economic loss estimated at USD 173 billion annually (Pires et al., 2022). Given the risks to both human health and agricultural sustainability, there is a growing demand for eco-friendly compounds that offer less harmful solutions for the environment and human wellbeing (Soliman et al., 2024). Natural products are emerging as compelling alternatives in the search for bioactive compounds. These molecules, originating from secondary metabolic processes in plants, fungi, bacteria, and animals, are synthesized in response to environmental stressors. In this context, natural products from the Andean region, obtained from fruits, medicinal plants, microorganisms, and lichens, represent an invaluable reservoir. Particularly, lichens, symbiotic associations of fungi with microalgae or cyanobacteria, are notable for their resilience to extreme habitats and capacity to produce bioactive secondary metabolites of significant pharmacological and agronomic relevance. Among the more than 19,000 documented lichen species, approximately 360 belong to the *Usnea* genus, which has been extensively investigated for its traditional uses in medicine, cosmetics, and food preservation (Lücking et al., 2017; Salgado et al., 2018). *Usnea lethariiformis* Motyka (Figure 1) occurs in Argentina and Chile and is widely distributed throughout the Andean Patagonian provinces. Its presence is mentioned in the Patagonian provinces of Tierra del Fuego, Santa Cruz, Río Negro, and Neuquén. *U. lethariiformis* is an exclusively corticolous species that is associated with forests and Patagonian shrub habitats. In very bright sectors, they usually occupy large areas in trunks and branches, together with *Protousnea* individuals (Rodriguez, 2011). Fanovich et al. (2013) have validated the efficacy of *U. lethariiformis* extracts in polycaprolactone–hydroxyapatite scaffolds against methicillin-resistant *Staphylococcus aureus* (MRSA) strains. The exploration of secondary metabolites within lichens has significantly advanced through ultra-high-performance liquid chromatography (UHPLC) coupled with mass spectrometry, a technique facilitating the precise analysis of diagnostic fragments specific to each compound (Torres-Benítez et al., 2017; Salgado et al., 2018; Albornoz et al., 2022; Areche et al., 2022). Notably, usnic acid and barbatic acid, major components in various *Usnea* species, have demonstrated activity against a broad range of pathogens and oxidant agents (Fanovich et al., 2016). Employing UHPLC-ESI-OT-MS-MS, up to 73 metabolites have been identified in methanolic extracts from species such as *U. barbata*, *U. antarctica*, *U. rubicunda*, and *U. subfloridana* (Salgado et al., 2018).

This study aims to provide a detailed chemical profile of *U. lethariiformis* extracts using UHPLC-ESI-Q-TOF-MS and to evaluate their trypanocidal, antimicrobial, antioxidant, and nematicidal activities. Exploring the metabolites could offer innovative solutions against hard-to-treat diseases and agricultural threats, marking an advancement in the sustainable use of Andean natural resources and biodiversity protection.

## 2 Material and methods

### 2.1 Chemicals

Ultra-pure water [<5 μg/L total organic carbon (TOC)] was obtained from an Arium 126 61316-RO water purification system plus an Arium 611 UV unit (Sartorius, Goettingen, Germany). Methanol (HPLC grade) and formic acid (puriss. p.a. for mass spectrometry) from J. T. Baker (Phillipsburg, NJ, USA) were obtained. Folin–Ciocalteu (FC) reagent, 2,2-diphenyl-1-picrylhydrazyl (DPPH), ferric chloride hexahydrate, 2,4,6-tris(2-pyridyl)-s-triazine, Trolox, quercetin, gallic acid, and DMSO with purity higher than 95% were purchased from Sigma-Aldrich Chem. Co. (St Louis, MO, USA) or Extrasynthèse (Genay, France). Cefotaxime was obtained from Argentia^®^ (Bristol-Myers Squibb, Buenos Aires, Argentina). Mueller–Hinton broth was provided by Laboratorio Britania (Buenos Aires, Argentina). Barbatic, divaricatic, divaricatinic, ursolic, and usnic acids, characterized previously by analysis of their spectroscopic data (^1^H and ^13^C NMR, MS), were used as standards for UHPLC-QTOF/MS/MS analysis. Some of the HPLC standards with a purity greater than 95% (stictic acid, diffractaic acid, barbatic acid, and usnic acid) were obtained either from Sigma (St. Louis, MO, USA), Biopurify (Chengdu, China), Phytolab (Vestenbergsgreuth, Germany), or Biosynth (Louisville, KY, USA) (Piovano et al., 2002; Schmeda-Hirschmann et al., 2008; Lima et al., 2012).

### 2.2 Collection of the lichen *Usnea lethariiformis*

The sample of *U. lethariiformis* was collected in April 2019 in the vicinity of the City of Tolhuin on both sides of RN3 (54°28′36″S 67°11′30″W); Tierra del Fuego, Argentina, as epiphytes on *Nothofagus pumilio* (*Fagaceae*) trees. This species was identified by the lichenologist Dr Juan Manuel Rodriguez, Universidad de Córdoba Argentina. A voucher specimen of samples has been deposited at the LUTI herbarium (LUTI-2404; LUTI-2405, Universidad Nacional de Córdoba, Argentina).

### 2.3 Dichloromethane (DCM) and methanol (MeOH) extracts

The air-dried *U. lethariiformis* (Ul) (180 g) material was extracted successively according to a previous methodology (Schmeda-Hirschamann et al., 2008) with DCM grade HPLC (2 × 24 h × 2 L) and MeOH grade HPLC (2 × 24 h × 2 L) at room temperature to afford UlDCM (5.5 g) and UlMeOH extract (15.6 g) with w/w yields of 2.77% and 8.66%, respectively. Both extracts were stored in a freezer at −40°C until their use for bioassays, phenolic identification/quantification, and UHPLC-ESI-QTOF-MS-MS analysis is given as Supplementary Material S1–S3.

The UlDCM extract (5 g) was treated with MeOH to afford an UlDCM MeOH-soluble (UlDCM-s) (2.75 g) fraction and an UlDCM MeOH-insoluble yellow precipitate (UlDCM-i) (2.25 g). Some 2.5 g of the bioactive UlDCM-s extract was applied to a Sephadex LH-20 column (length 33 cm, diameter 5.5 cm) eluted with petroleum ether (PE), DCM and MeOH (2:1:1 v:v). Some 15 fractions of 50 mL each were obtained. After TLC comparison (silica gel, PE:EtOAc, 8:2 v:v as the mobile phase; detection under UV light and after spraying with p-anisaldehyde) fractions with similar TLC patterns were combined affording the fraction pools 1 (23.1 mg; fraction 1), 2 (203.1 mg, fraction 2), 3 (165.1 mg, fraction 3), 4 (97.3 mg, fraction 4), 5 (40.6 mg, fraction 5), 6 (75.9 mg, fraction 6), 7 (120.9 mg, fraction 7); 8 (237 mg, fraction 8), 9 (109.9 mg, fraction 9) and 10 (24.5 mg, fractions 10–15).

### 2.4 Ultra-high performance liquid chromatography analysis (UHPLC-ESI-QTOF-MS)

The separation and identification of secondary metabolites from lichen extracts were carried out on a UHPLC-ESI-QTOF-MS system equipped with UHPLC Ultimate 3000 RS with Chromeleon 6.8 software (Dionex GmbH, Idstein, Germany) and a Bruker maXis ESI-QTOF-MS. The chromatographic equipment consisted of a quaternary pump, an autosampler, a thermostatted column compartment, and a photodiode array detector. The elution was performed using a binary gradient system with eluent (A) 0.1% formic acid in the water, eluent (B) 0.1% formic acid in acetonitrile, and the gradient: isocratic 1% B (0–2 min), 1%–5% B (2–3 min), isocratic 5% B (3–5 min), 5%–10% B (5–8 min), 10%–30% B (8–30 min), 30%–95% B (31–38 min), and 1% B isocratic (39–50 min). The separation was carried out with an acclaim Thermo 5 µm C18 80 Å (150 mm × 4.6 mm) column at a flow rate of 1.0 mL/min. ESI-QTOF-MS experiments in negative ion mode were recorded, and the scanning range was between 100 m/z and 1,200 m/z. Electrospray ionization (ESI) conditions included a capillary temperature of 200°C, a capillary voltage of 2.0 kV, a dry gas flow of 8 L/min, and a pressure of 2 bars for the nebulizer. The experiments were performed in the automatic MS/MS mode.

The annotation of the bioactive compounds was based on HR full MS, fragmentation patterns, and similarity with literature data. For the analysis, 5 mg of each extract was dissolved in 2 mL of methanol, passed through a polytetrafluoroethylene (PTFE) filter, and 10 µL was injected into the apparatus. To double check for identity, a solution of 500 μg/mL of each available standard was prepared. Of this solution, 10 μL was co-injected and checked for HPLC-DAD chromatogram changes. MS data were analyzed using Bruker Data Analysis 4.0 (Bruker Daltonik GmbH, Bremen, Germany) and ACD lab spectrum processor (New York, USA) software. Supplementary Figure S1 shows an HPLC-DAD chromatogram (Thermo Dionex 3000 RS) of a crude methanolic extract of *U. lethariiformis* (UlMeOH), Supplementary Figure S2 shows thechromatogram at 280 nm of the pure standard of usnic acid and its DAD spectra, and Supplementary Figure S3 shows the MS and MSn spectra of lichenic compounds.

### 2.5 Total phenolic content (TP)

The total phenolic content of UlDCM and UlMeOH was determined by the Folin–Ciocalteu reagent in a microplate (Gómez et al., 2021). The total phenolics were expressed as milligrams of gallic acid equivalents (GAE) per gram of extract (mg GAE/gUlDCM-s or UlMeOH). The values were obtained using a Multiskan FC Microplate Photometer (Thermo Scientific, Waltham, MA, USA) and are shown as the mean ± standard deviation (SD).

### 2.6 Trypanocidal activity

#### 2.6.1 *Trypanosoma cruzi* cultures

*T. cruzi* epimastigotes (Dm28c strain) were cultured at 28°C in Diamond medium (0.1 M NaCl, 0.05 M K_2_HPO_4_, 0.625% tryptose, 0.625% tryptone, 0.625% yeast extract, pH 7.2) supplemented with 10% inactivated fetal bovine serum (Gibco) and 12.5 μg/mL hemin (Piñeiro et al., 2023).

#### 2.6.2 Proliferation

Epimastigotes (adjusted to 3 × 10^6^ cells/mL) were incubated at 28°C with different concentrations (10–100 μg/mL) of UlDCM, UlMeOH, or UlDCM-s Sephadex LH-20 fractions in sterile tubes, and the volume was adjusted to 1 mL with Diamond medium. Controls without compounds with 0.2% of the diluent DMSO or benznidazole (19.2 μM) were used in all experiments. Aliquots were collected every 24 h and were fixed with 2% p-formaldehyde in PBS. Parasites were counted in a Neubauer hemocytometer (Piñeiro et al., 2023).

### 2.7 Antibacterial activity

#### 2.7.1 Microorganisms

For antimicrobial evaluation, strains from the American Type Culture Collection (ATCC, Rockville, MD, USA) and clinical isolates from Laboratorio de Microbiología, Hospital Marcial Quiroga, San Juan, Argentina (MQ), were used. The panel comprised the following bacteria: *S. aureus* methicillin-sensitive ATCC 29213, *S. aureus* methicillin-resistant ATCC 43300, *S. aureus* methicillin-resistant-MQ1, *S. pyogenes*-MQ4, *E. coli* ATCC 25922, and *Salmonella* sp. The yeasts *Candida albicans* 1792, *C. tropicalis*-MQ1, and *C. parapsilopsis*-MQ-1 were also used.

#### 2.7.2 Antibacterial susceptibility testing

The antibacterial activity was evaluated following the protocol proposed by the Clinical and Laboratory Standards Institute (CLSI) for the microdilution assay in Mueller–Hinton broth (CLSI, 2013). The UlDCM and UlMeOH extracts and UlDCM-s fraction were tested from 100 μg/mL to 1,000 μg/mL using the inoculum of each bacterium adjusted to 5 × 10^5^ cells with colony-forming units (CFU)/mL). Meropenem was used as a reference compound (2 μg/mL). The absorbances at 620 nm were determined in a Multiskan FC Microplate Photometer (Thermo Scientific, Waltham, MA, USA), and the inoculated microplates were incubated at 37°C. Subsequently, minimum inhibitory concentration (MIC in µg/mL) was defined as the lowest extract/fraction concentration showing no visible bacterial growth after incubation time. The minimum bactericidal concentration (MBC in µg/mL) was determined by taking 10 µL from the well without bacterial growth, and it was added to a new sterile microplate with culture medium. Evidence of growth was determined after incubation for 24 h. The MBC was defined as the lowest concentration of each compound that resulted in total inhibition of visible growth in these plates.

#### 2.7.3 Antifungal susceptibility testing

The effect of the UlDCM and UlMeOH extracts and the UlDCM-s fraction against the yeasts was carried out by broth microdilution techniques (CLSI, 2002). The samples were diluted in DMSO in the assay (≤1%). The inoculums of 24 h were adjusted with sterile physiological solutions to give a final organism density of 5 × 10^4^ CFU/mL. The reference compound used was ketoconazole (2 μg/mL). The microplates were incubated at 28–30°C, and the absorbances were measured at 405 nm. The results were expressed as MIC (µg/mL) and MBC (µg/mL) when the samples showed activity.

### 2.8 Nematicidal activity

#### 2.8.1 Nematode populations

*M. incognita* populations were collected from a susceptible tomato cultivar (*Solanum lycopersicum*) from fields of Rawson, San Juan province, Argentina. Subsequently, this population was reared on tomato seedlings in a pot laboratory. After 40 days, infected roots were collected and washed gently with tap water. Under a stereoscopic microscope at ×1.6, egg masses were hand-picked and put in 1% NaOCl solution for 4 min to dissolve the gelatinous matrix. They were incubated in a growth chamber at 28 ± 1°C. After hatch, second-stage juveniles (J2) were collected up to 3 days old for the assays (Manrique et al., 2023).

#### 2.8.2 Nematicidal assay

Glass Petri dishes of 50 mm ø were used, where 20 J2s were placed on 10 mL of the solution to be evaluated. Five replicates per treatment were performed. From each Ul extract (UlMeOH, UlDCM-s, and UlDCM-i), 50 mg was weighed in an Eppendorf tube, to which 1 mL of MeOH and 300 µL of Tween 20 were added. The suspension was sonicated for 30 min. Then, the flask was made up to 100 mL of solution with filtered H_2_O. The final concentration of this solution was 0.05% w/v. For the control of solvents, methanol and Tween-20 in filtered H_2_O were used in identical proportions without the addition of the extracted sample. A solution with the solvent used in each treatment was prepared at the concentration used in the assay as a negative control. As a positive control, abamectin (Abamex^®^, Nufarm, Argentina) diluted in water was used at the concentration indicated by the manufacturer (0.0018% v/v). The dishes were randomly distributed on a tray and taken to an incubation chamber at 28°C in the dark.

Counts of immobile individuals were made every 24 h, 48 h, and 72 h. After 72 h, the immobile individuals were placed in filtered water to confirm their mortality (Manrique et al., 2023).

Statistical analysis: the data of immobile individuals per hour were expressed as a percentage, and two-way ANOVA was applied (treatment, hours and interaction). When the factors were significant (*p* < 0.05), the statistical difference between means was determined according to Fisher’s least significant differences (LSD) test, partitioned by hours, using the Infostat/L 2020 software. Mortality data for J2 per treatment were expressed in percentages and were used to calculate the treatment efficiency (ET) by applying the Schneider–Orelli formula, which discounts the natural mortality of the control from the mortality of the treatment:

$$\text{ET }\left(\%\right)=\frac{\text{MT}-\text{MCO}}{100-\text{MCO}}\;*100.$$

Schneider–Orelli treatment efficiency formula, where ET: treatment efficiency expressed as a percentage, MT: treatment mortality expressed as a percentage, and MCO: control mortality expressed as a percentage.

The ET data were submitted to a one-way analysis of variance (ANOVA) (treatment), and Fisher’s LSD test was used (*p* < 0.05) to determine the statistical differences between the means using the Infostat/L software (2020).

To assess the data, the scale proposed by Faria et al. (2021), which classifies the nematicidal activity as total (100%), high (99%–80%), moderate (79%–60%), weak (59%–40%), and low (< 39%).

The LC_50_ and its confidence intervals were estimated using a sigmoid dose–response model, using R version 4.3.3 and the “drc” library.

### 2.9 Antioxidant activity

#### 2.9.1 Radical scavenging capacity assay of 2,2-diphenyl-1-picrylhydrazyl

The extracts’ radical scavenging capacity to 2,2-diphenyl-1-picrylhydrazyl (DPPH) was determined using the following procedure: the DPPH solution (20 mg/L) in methanol was mixed with the extract solution at concentrations between 1 μg and 1,000 μg/mL (Gómez et al., 2021). The reaction progress absorbance of the mixture was monitored at 515 nm using a Multiskan FC Microplate Photometer (Thermo Scientific, Waltham, MA, USA). The percentage of the DPPH de-coloration was proportional to the five antioxidant concentrations, and the concentration responsible for a decrease in the initial DPPH concentration by 50% was defined and calculated as EC_50_ value (mean ± SD).

### 2.10 Inhibition of lipid peroxidation in erythrocytes

The ability of the extracts to inhibit tert-butyl hydroperoxide-induced lipoperoxidation in erythrocytes (LP) at three concentrations (100 μg, 2500 μg, and 500 μg/mL) and of catechin at a single concentration (100 μg/mL) was determined. Relevant technical aspects of the trial have been reported recently in detail (Gómez et al., 2021). The values obtained were calculated as percentages of lipid oxidation inhibition (ILP).

## 3 Results and discussion

### 3.1 Phenolics contents and UHPLC-ESI-QTOF-MS-MS analysis

The UlDCM-s and UlMeOH extracts showed phenolic content of 107 mg and 48 mg GAE/g dried extract, respectively. Despite the significant content of phenolic compounds, the UlDCM-s, UIDCM-i, and UlMeOH extracts showed a moderated free radical scavenging activity in the DPPH, FRAP, and TEAC until 1 mg/mL, and in the ILP tests at 250 μg/mL (data not shown).

The use of HPLC or UPLC coupled to hybrid state-of-the-art mass spectrometers, such as tandem quadrupole mass spectrometers (TQ), quadrupole time-of-flight (Q-TOF), or quadrupole-Orbitrap^®^ (Q-OT), provides fast and reliable data analysis of phenolics compounds in plant samples and other biological matrices. A considerable number of Andean species, including lichen extracts, have been recently thoroughly analyzed using this technology (Salgado et al., 2018; Gómez et al., 2019a; 2019b; 2020; 2021; Barrientos et al., 2023; Manrique et al., 2023), and their chemical composition has been updated. Until now, there were no reports about chemical characterization using UHPLC/MS/MS and the trypanocidal and nematicidal activities of the dichloromethane and methanol extracts (UlDCM-s, UlDCM-i, and UlMeOH) obtained from this species.

**FIGURE 2:**
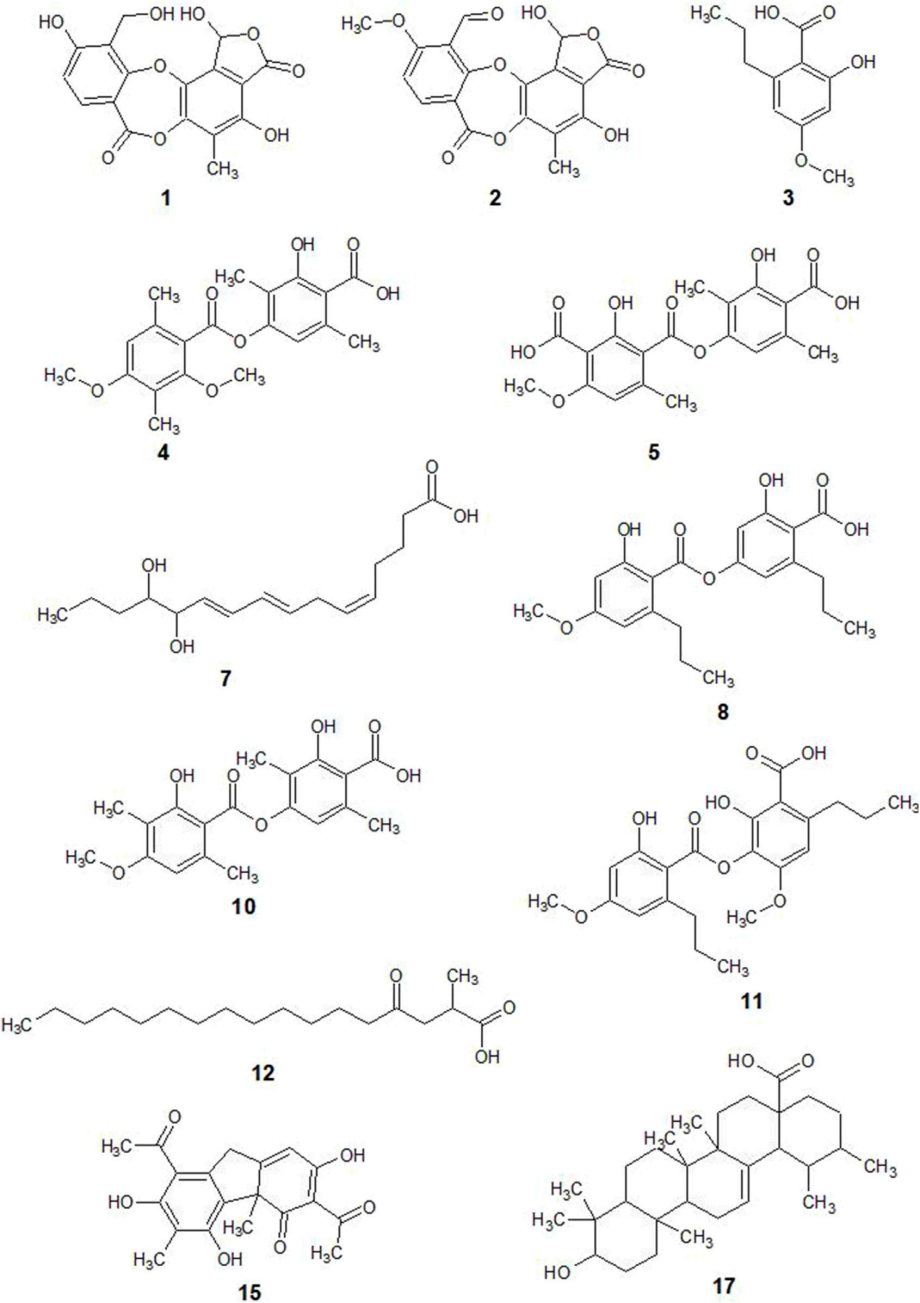
Chemical structures of compounds identified in *Usnea lethariiformis* extracts.

**FIGURE 3:**
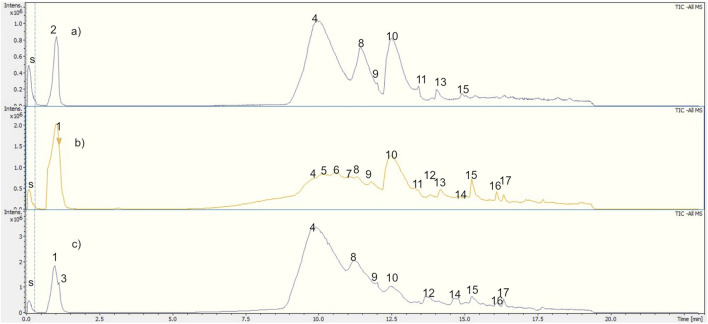
Total ion chromatograms (Bruker QtoF-MS) of **(A)** UlDCM-s, **(B)** UlDCM-i, and **(C)** UlMeOH extracts from *U. lethariiformis*. Internal standard: sodium formiate.

The main phenolic compounds in the lichen were detected in UlDCM and UlMeOH extracts by UHPLC-ESI-Q-TOF-MS-MS analysis. Metabolite assignments were made combining full mass spectra and MS^2^ experiments (accurate mass and fragmentation pattern), retention times, comparison to the standard compounds, and search in public online-databases (LDB Lichen Database, on the Global Natural Product Social Molecular Networking (*GNPS*) site, Mona MassBank of North America, Reaxys and SciFinder), respectively. Seventeen compounds were rapidly identified in UlDCM-s, UlDCM-i, and UlMeOH extracts using available standards and databases (Schmeda-Hirschmann et al., 2008; Salgado et al., 2018; Olivier-Jimenez et al., 2019). Recently, the chemical fingerprinting analysis of secondary metabolites, including barbatic acid, diffractaic acid, divaricatic acid, and usnic acid, from four *Usnea* species (*Usnea barbata, Usnea antarctica, Usnea rubicunda*, and *Usnea subfloridana*) was reported using hyphenated techniques (Salgado et al., 2018). Table 1 and Figures 2, 3 show the identification of metabolites in *Usnea lethariiformis* by UHPLC-ESI-QTOF-MS-MS. The metabolomics identification is explained below in detail. Additional information about the UHPLC-ESI-QTOF-MS-MS analysis is given in Supplementary Materials S1–S3.

**TABLE 1:** Identification of metabolites in *Usnea lethariiformis* by UHPLC-ESI-QTOF-MS-MS.

RT (min)	Tentative identification	Molecular formula	Measured mass (m/z)	Ion MS^2^	Extract
0.9	Connorstictic acid **(1)**	C_18_H_14_O_9_	373.1008	373.1693 (27); 329.1045 (100); 314.0773 (29); 285.1093 (29); 270.0801 (49); 226.0871 (35); 181.0655 (43); 163.0505 (29); 151.0521 (58)	UlDCM-i
1.0	Stictic acid^a^ **(2)**	C_19_H_14_O_9_	385.0996	385.1035 (14); 343.1185 (19); 341.1082 (100); 299.1239 (26); 297.1084 (98); 282.0807 (32); 269.1099 (14); 267.0568 (42); 255.0896 (14); 254.0838 (25) 253.0758 (12); 239.0586 (15); 238.0899 (23); 226.0855 (24); 225.0810 (48); 197.0796 (41); 149.0329 (13)	UlDCM-s
1.1	Divaricatinic acid **(3)**	C_11_H_14_O_4_	209.1019	209.0981 (7); 165.1036 (73); 150.0782 (100); 122.0413 (46); 93.0313 (6)	UlDCM-s; UlDCM-i
9.8	Diffractaic acid^a^ **(4)**	C_20_H_22_O_7_	373.1711	769.3245, (50) 373.1714 (100); 329.1764 (33); 297.1459 (24); 209.1024 (19); 135.0874 (16); 120.0606 (7)	UlDCM-s; UlDCM-i; UlMeOH
9.9	Squamatic acid **(5)**	C_19_H_18_O_9_	389.1676	225.0975 (35); 181.0656 (100); 166.0754 (34); 137.0665 (25); 122.0403 (34)	UlDCM-i
10.0	4-O-demethyldivaricatic acid **(6)**	C_18_H_14_O_9_	373.1717	769.3266 (63); 373.1717 (100); 329.1770 (34); 297.1462 (29); 285.1808 (10); 209.1019 (28); 135.0877 (9)	UlDCM-i
10.1	Dihydroxyheptadecatrienoic acid **(7)**	C_17_H_28_O_4_	295.2623	295.2624 (100); 277.2493 (56); 195.1585 (13); 183.1561 (45); 171.1170 (15)	UlDCM-i
11.3	Divaricatic acid **(8)**	C_21_H_24_O_7_	387.1890	195.0841 (100); 177.0704 (80); 151.0863 (37); 133.0717 (18); 109.0665 (8); 91.0148 (11)	UlDCM-s; UlDCM-i; UlMeOH
11.6	Unknown **(9)**	C_21_H_24_O_7_	797.3601	797.3601 (32); 605.2703 (22); 413.1679 (8); 387.1883 (9); 195.0835 (100); 177.0705 (57); 151.0857 (8)	UlDCM-s; UlDCM-i; UlMeOH
12.5	Barbatic acid^a^ **(10)**	C_19_H_20_O_7_	359.1546	181.0659 (100); 163.0521 (39); 137.0677 (30); 119.0532 (14); 93.0674 (6)	UlDCM-s; UlDCM-i; UlMeOH
12.9	Sekikaic acid **(11)**	C_22_H_26_O_8_	417.2033	225.0997 (40); 209.1023 (100); 166.0765 (48); 165.1050 (29); 150.0783 (33); 137.0313 (13); 122.0409 (13)	UlDCM-s; UlDCM-i
12.8	Lichesterylic acid **(12)**	C_18_H_34_O_3_	297.2765	297.2772 (100); 279.2601 (12); 155.1177 (8)	UlDCM-1; UlMeOH
12.9	Unknown **(13)**	-	563.2167	563.2167 (17); 359.1546 (15); 181.0662 (100); 163.0523 (39) 137.0682 (8)	UlDCM-s; UlDCM-i
13.5	Unknown **(14)**	-	1,053.3244	1,053.3244 (43); 343.1208709.2185 (42); 694.1927 (35); 343.1208 (100); 259.0885 (21); 231.0900 (20)	UlDCM-i; UlMeOH
13.7	Usnic acid^a^ **(15)**	C_18_H_16_O_7_	343.1214	343.1216 (73); 328.0959 (100); 313.0707 (18); 299.1263 (13); 259.0890 (71); 257.0731 (15); 231.0897 (20)	UlDCM-s; UlDCM-i; UlMeOH
13.8	Unknown **(16)**	-	259.0891	259.0891 (100); 231.0901 (8); 83.0075 (6)	UlDCM-i; UlMeOH
14.7	Ursolic acid **(17)**	C_30_H_48_O_3_	455.4047	455.4088 (100)	UlDCM-i; UlMeOH

^a^ identified by co-spiking with authentic standards.

#### 3.1.1 Depsidones

Two depsidones corresponding to compounds 1 and 2 were identified using UHPLC-ESI-QTOF-MS-MS analysis. Compound 1 was identified as connorstictic acid, which showed an [M - H]^−^ ion at m/z 373.1008. Its major diagnostic daughter ions were at m/z 329.1045, 181.0655, and 151.0521. Compound 2 with an [M - H]^−^ ion at m/z 385.0996 was identified as stictic acid, which showed diagnostic daughter ions at m/z 341.1082, 297.1084, 267.0568, 225.0810, and 197.0796 confirming this depsidone. These findings are in good agreement with the reports of the lichen species (Salgado et al., 2018; Le Pogam et al., 2015; Musharraf et al., 2015; Castro et al., 2017).

#### 3.1.2 Depsides

Six depsides were identified (compounds 4, 5, 6, 8, 10, and 11). Compound 4 was identified as diffractaic acid (molecular anion at m/z 373.1711), whose fragmentation produced diagnostic MS^2^ ions at m/z 329.1764, 297.1459, and 135.0874. Compound 6 was tentatively identified as 4-O-demethyldivaricatic acid, showing a [2M - 2H + Na]^−^ ion at m/z 769.3276 and an MS ion at m/z 373.1717. Compound 5 was identified as squamatic acid, which showed an [M − H]^−^ ion at m/z 389.1676. Compound 8 was identified as divaricatic acid (m/z = 387.189). Compound 10 was identified as barbatic acid (m/z = 359.1546). Its major diagnostic daughter MS ions were at m/z 181.0659, 163.0521, and 137.0677. Compound 11 was identified as sekikaic acid, whose molecular anion was at m/z 417.2033. These findings are in good agreement with the reports of Salgado et al. (2018) on species belonging to the *Usnea* genus.

#### 3.1.3 Fatty acids

Two fatty acids were identified using UHPLC-ESI-QTOF-MS-MS analysis. Compounds 7 and 12 presented an [M - H]^−^ ion at m/z 295.2623 and 297.2765, respectively, which were tentatively identified as dihydroxyheptadecatrienoic acid and lichesterylic acid, respectively. Compound 7 was previously reported in *U. barbata* (Salgado et al., 2018). However, to the best of our knowledge, it is the first report of lichesterylic acid (12) in the genus *Usnea*.

#### 3.1.4 Dibenzofurans

Peak 15 was unequivocally identified as usnic acid, using a sample previously isolated as a reference standard (Schmeda-Hirschamann et al., 2008) and validated for its spectroscopic properties (^1^H and ^13^C NMR, MS) showing an [M - H]^−^ ion at m/z 343.1214. The main daughter ions were [M - H - CH_3_]^−^, [M - H - C_4_H_3_O_2_]^−^, and [M - H - C_5_H_3_O_3_]^−^ (m/z 328.0959, 259.0890, and 231.0897, respectively).

#### 3.1.5 Other compounds

Finally, a benzoic acid (3) and a triterpene (17) corresponding to divaricatinic acid and ursolic acid were identified in this extract. Four additional compounds were detected that have yet to be identified (9, 13, 14, and 16).

### 3.2 Effects on the proliferation of *T. cruzi* epimastigotes

According to the World Health Organization (2023), approximately six to seven million people are infected with the protozoan *T. cruzi*, the etiological agent of Chagas disease, which is endemic to 21 countries in Latin America. Chagas disease is transmitted to humans primarily through contact with feces or urine from triatomine bugs (vector-borne transmission), also known as kissing bugs, among many other popular names, depending on the geographical area.

Benznidazole and nifurtimox showed high efficiency in curing Chagas disease, including cases of congenital transmission. However, the efficacy of both drugs diminishes the longer a person has been infected, and the adverse reactions are more frequent at older age. This supports the need to continue searching for new chemical identities with trypanocidal potential in underexplored natural sources such as the flora of Argentina.

**FIGURE 4:**
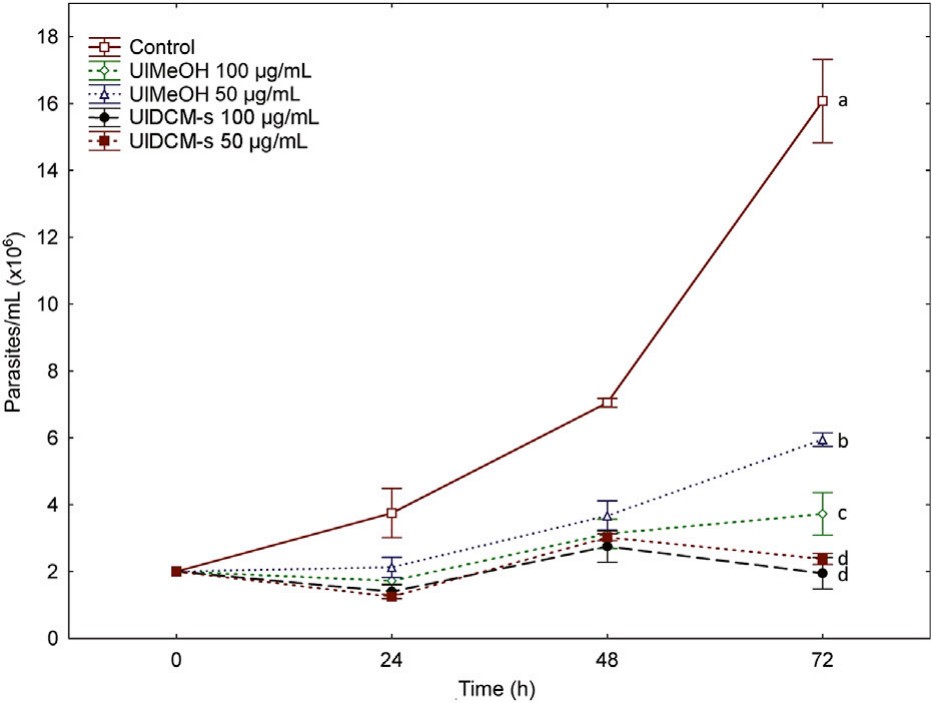
Effects of UlMeOH and UlDCM-s against *T. cruzi* epimastigotes. Different letters indicate significant differences (*p* < 0.05).

The interest in the search for new bioactive molecules in lichen species has increased in recent years due to the quantity and diversity of compounds they produce. In this sense, UlMeOH, UlDCM-i, and UlDCM-s Sephadex fractions were evaluated. The biological activity against *T. cruzi* epimastigotes of UlMeOH and UlDCM-s was evaluated at two concentrations (50 μg/mL and 100 μg/mL, Figure 4). Both UlMeOH and UlDCM-s extracts showed activity with significant differences, with respect to the control, in the two concentrations evaluated. These differences were maintained during the three tests. The treatments with UlDCM-s were the ones that presented the greatest activity.

Given these results, UlDCM-s Sephadex fractions were evaluated at 10 μg/mL and 40 μg/mL against *T. cruzi* epimastigotes. The percentage of proliferation inhibition is presented in Table 2. The inhibition of proliferation was dose-dependent, the 40 μg/mL fractions being the most inhibitory with respect to the 10 μg/mL fractions. Likewise, fraction 5 was the only one that showed a significant inhibition, with respect to the control, in its two concentrations during 24 h, 48 h, and 72 h. On the other hand, for the concentration of 40 μg/mL, fractions 4, 6, and 7 and usnic acid showed significant differences, with an inhibition close to 100%.

**TABLE 2:** *In vitro* activity of UlDCM-s Sephadex fractions against *T. cruzi* epimastigotes.

Fraction/compound	Concentration (µg/mL)	% growth inhibition		
		24 h	48 h	72 h
*Fraction2*	10	1.09 ± 0.03	2.14 ± 0.65	3.68 ± 0.41
	40	35.59 ± 2.23	38.71 ± 4.21	44.62 ± 3.67
*Fraction4*	10	15.82 ± 1.87	0.21 ± 0.11	27.05 ± 2.47
	40	31.64 ± 0.55	87.82 ± 3.77*	98.16 ± 0.98*
*Fraction5*	10	52.59 ± 4.89*	57.48 ± 4.23*	77.62 ± 2.33*
	40	92.09 ± 2.44*	95.73 ± 2.19*	99.15 ± 1.03*
*Fraction6*	10	26.31 ± 3.89	29.66 ± 2.73	47.82 ± 0.84
	40	94.53 ± 3.29*	97.56 ± 1.16*	98.24 ± 1.26*
*Fraction7*	10	24.29 ± 1.13	25.64 ± 4.03	46.6 ± 3.78
	40	95.48 ± 2.07*	98.50 ± 0.72*	97.31 ± 2.77*
*Fraction9*	10	0.56 ± 0.06	2.56 ± 0.99	4.25 ± 1.54
	40	35.82 ± 1.96	39.23 ± 3.08	43.34 ± 3.72
*Usnic acid*	10	18.98 ± 2.81	31.2 ± 2.10	52.55 ± 4.13
	40	77.97 ± 3.39*	91.67 ± 2.51*	98.19 ± 0.79*

* indicates significant differences compared to the control.

Remarkable reports on the chemical composition and biological activities of the species of the genus *Usnea*, as well as the biological activities of secondary metabolites identified in the species, have been published in the last decade (Sepahvand et al., 2021). The antimicrobial activity of extracts and metabolites from species belonging to the genus *Usnea* against bacteria, fungi, protozoa, and viruses stands out in the evaluated activities. The strong antimicrobial activity of *Usnea subflorida* correlated with their high content of usnic acid has been reported (Cansaran et al., 2006). Similarly, antibacterial, antimycotic, and antiprotozoal activity of DCM and MeOH extracts and the major metabolites, such as usnic and isodivaricatic acid, of *Usnea florida* has been reported (Schmeda-Hirschmann et al., 2008).

Regarding *U. lethariiformis*, the depsides, divaricatic acid, barbatic acid, and diffractaic acid, as well as the dibenzofurans usnic acid, were identified by UHPLC-ESI-QTOF-MS-MS analysis of UlDCM and UlMeOH extracts and showed potent trypanocidal activity. From them, the trypanocidal effect of usnic acid isolated from lichen *Cladonia substellata* against *T. cruzi* has been reported (De Carvalho et al., 2005). They observed widespread ultrastructural damage in *T. cruzi* treated with usnic acid, including mitochondrial injury, with a marked increase in kinetoplast volume and vacuolation of the mitochondrial matrix, intense lysis, and enlargement of the flagellar pocket. On the other hand, the host cell showed no significant ultrastructural damage (De Carvalho et al., 2005). Moreover, the trypanocidal effect of the barbatic acid isolated from lichen *Cladonia salzmannii* was reported to epimastigotes of *T. cruzi* (Vasconcelos, 2005). Barbatic acid at concentrations of 20 μg/mL and 40 μg/mL showed significant inhibition of proliferation and morphological changes, as well as induction of aberrant-shaped cells and abnormal mitosis process with displaced organelles (Vasconcelos, 2005). Ina previous report, isodivaricatic acid, divaricatic acid, and usnic showed a stronger activity towards *Leishmania amazonensis*, *L. brasiliensis*, and *L. infantum* promastigotes with 100% lysis at 100 μg/mL (Schmeda-Hirschmann et al., 2008). Recently, the strong activity antiparasitic of usnic acid against *L. major*, *L. infantum*, and *L. tropica* promastigotes with an IC_50_ between 10.76 μg/mL and 21.06 μg/mL was reported (Derici et al., 2018). These reports support that the trypanocidal activity of UlDCM and UlMeOH extracts and UlDCM Sephadex fractions would be given by the presence of at least two compounds (usnic and barbatic acids).

Biodiversity in Argentina emerges as a fundamental source of extracts, pure compounds, and derivatives with notable efficacy against *T. cruzi*, according to various studies. Schmeda-Hirschmann et al. (2008) revealed a novel depside with antiprotozoal properties extracted from the Andean lichen *Protousnea poeppigii*. Similarly, Lozano et al. (2016) explored the diterpene 5-epi-icetexone from *Salvia gilliesii*, showcasing its marked *in vivo* trypanocidal effect. Furthermore, Spina et al. (2018) conducted a comprehensive analysis of the antiproliferative effects and ultrastructural alterations induced by 5-O-methylembelin, a derivative of embelin obtained from *Oxalis erythrorhiza*. Recent investigations, such as those by Piñeiro et al. (2023), highlighted the potent antiparasitic activity of *Hippeastrum* species, emphasizing the synergistic interaction between the alkaloid montanine and benznidazole. Additionally, alkaloids isolated from *Amaryllidaceae* plants in Argentina, such as ismine and candimine, demonstrated significant efficacy against *T. cruzi*, affecting both replicative and non-replicative forms (Martinez-Peinado et al., 2022; Ortiz et al., 2023).

These collective findings not only underscore the extraordinary biological diversity present in Argentina but also offer promising perspectives for the development of more effective treatments against Chagas disease, among other diseases with a strong impact on human health, such as infections due to resistant bacteria and fungi, parasites, diabetes, and cancer, for which new bioactive chemical structures are in high demand.

Recently, studies of lichens from Antarctica and other Andean regions as sources of antioxidant, cytoprotective, anti-inflammatory, and enzyme-inhibiting compounds with an impact on human health have been reported, which support the need for further studies of the chemistry and biological activity of these natural products, many of which grow in extreme climatic conditions (Torres-Benítez et al., 2022; Torres-Benítez et al., 2023a; Torres-Benítez et al., 2023b).

### 3.3 Antimicrobial activity

The UlDCM-s, UlDCM-i, and UlMeOH extracts and Sephadex fraction from UlDCM-s were assayed for antibacterial and antifungal properties with the microbroth dilution method following the guidelines of CLSI at concentrations between 1 μg/mL and 500 μg/mL against a panel of standardized strains and clinical isolates from the Marcial Quiroga Hospital, located in the Province of San Juan, Argentina. Results (Table 3) show that all bacteria and yeasts tested were inhibited by the different extracts with MIC values between 25 μg/mL and 500 μg/mL. The panel of fungi included the yeasts *C. albicans*, *C. tropicalis*, and *C. parapsilosis*.

**TABLE 3:** Antimicrobial activity of *Usnea lethariiformis* extracts [minimum inhibitory concentrations (MICs) and minimum bactericidal/fungicidal concentrations (MBCs/MFCs) in µg/mL].

Bacteria/Yeast	UlDCM-i	UlDCM-s	UlMeOH	UlDCM-s Sephadex fractions			Usnic acid	Cefo	Imi	Mero	Ket
				F5	F6	F7					
	MIC/MBC/MFC									
MSSA	25/250	62.5/500	125/>500	25/50	25/25	25/100	25>100	0.5/0.5	0.5/0.5	2/>2	-
MRSA	125/125	125/125	125/125	25/>200	25/200	25/200	12.5>100	0.5/0.5	0.5/0.5	0.25/>2	-
MRSA-MQ1	12.5/1000	50/>500	125/125	25/>200	25/>200	25/>200	12.5>100	0.8/1	1/1	2/>2	-
*S. pyogenes* -MQ4	125/125	50/250	125/125	25/>250	25/>100	25/>100	25/25	0.25/0.5	0.5/0.5	>2	-
*E. coli* ATCC 25922	>1000	>1000	>1000	>200	>200	>200	>100	1.9/1.9	0.5/1	0.5/1	-
*C. albicans*	125/250	125/250	125/250	50/100	50/100	50/100	25/50	-	-	>5	>5
*C. tropicalis*	500/>500	125/>250	125/500	100/>200	50/>200	50/100	25/50	-	-	1.25/5	1.25/5
*C. parapsilosis*	>500	500/>500	>500	>200	200/>200	100/100	>100	-	-	>100	0.62/0.62

MSSA: methicillin-sensitive *S. aureus* ATCC 25923; MRSA: methicillin-resistant. *S. aureus* ATCC 43300; MIC: minimum inhibitory concentration, MBC: minimum bactericidal concentration. Cefo: cefotaxime; Imi: imipecil; Mero: meropenen; Ket: ketoconazole.

The clinical yeast *C. albicans* was significantly inhibited, with MIC/MBC values of 125 μg/mL. Interestingly, Sephadex fractions 6 and 7 of UlDCM-s were very fungistatic and fungicidally active, with low MIC/MFC values against *C. albicans* (50/100 μg/mL). The same fractions were very active against the yeast *C. tropicalis*, with MIC/MFC values of 50/200 μg/mL. According to De Tamokou et al. (2017a), an extract is very active if MIC values <100 μg/mL, significantly active if 100 ≤ MIC ≤512 μg/mL, moderately active if 512 μg/mL ≤MIC ≤2048 μg/mL, and not very active if MIC >2048 μg/mL. Regarding *C. parapsilosis*, only the UlDCM-s was significantly active with MIC/MFC of 500/˃500 μg/mL.

Natural products play an important role in the development of drugs for the treatment of human diseases. In recent decades, the medicinal and non-medicinal flora of the Andes has been exhaustively analyzed using standardized techniques according to CLSI or EUCAST standards to discover plant extracts, essential oils, compounds, ash semisynthetic derivatives with antimicrobial activities against MSSA, MRSA, and yeast, including *Cryptococcus, Candida* (*C. albicans* and non-albicans *Candida* spp.), and dermatophyte strains (Lima et al., 2015; Butassi et al., 2019; Dolab et al., 2018; Martin-Acosta et al., 2020). *Candida* and *Cryptococcus* strains are relevant fungi involved in the epidemiology of fungal infections. *Candida* spp. are among the leading causes of nosocomial blood stream infections worldwide, and although *C. albicans* was in the past associated with invasive mycoses, at present non-albicans *Candida* spp. (*C. tropicalis*, *C. glabrata*, *C. parapsilopsis*, *C. krusei,* and others) comprise more than half of the isolates of candidiasis in immune-compromised hosts (Almirante et al., 2005).

Recently, *Candida auris*, an emerging fungus considered an urgent resistance threat to echinocandins, the most recommended antifungal drug for the treatment of *C. auris* infections, spread at an alarming rate in US healthcare facilities in 2020–2021, according to data from the Centers for Disease Control and Prevention. *C. auris* is a multidrug-resistant yeast that can cause invasive infection and death. It spreads easily among hospitalized patients and nursing home residents. Particularly concerning, some strains of *C. auris* are resistant to all three antibiotic classes used to treat fungal infections. These data show that additional action, including the search for new antimicrobials, is critical to slow the spread and impact of antimicrobial resistance (https://www.cdc.gov/2019; https://www.who.int/2022).

Methicillin-resistant *S. aureus* (MRSA) is one of the leading causes of infections such as bacteremia, pneumonia, endocarditis, and complicated skin and soft-tissue infections presenting in healthcare and community settings. Such infections are associated with high morbidity and mortality because of their persistent outbreaks and poor clinical outcomes (Liang et al., 2022).

The UlDCM-i, UlDCM-s, and UlMeOH extracts showed a range of MICs between 25 μg/mL and 125 μg/mL against MSSA and a significant activity against MRSA with MICs/MBC equal to 125/125 μg/mL. The UlDCM-i and UlDCM-s displayed a very strong activity toward MRSA-MQ1, showing MIC values between 25 μg/mL and 125 μg/mL. On the other hand, the UlDCM-s Sephadex fraction showed a very bacteriostatic activity again MSSA, MRSA, and the isolate MRSA-MQ1 with MICs = 25 μg/mL. Fanovich et al. (2013) validated the efficacy of *U. lethariiformis* extracts in polycaprolactone-hydroxyapatite scaffolds against methicillin-resistant *S. aureus* (MRSA) strains. According to De Tamokou et al. (2017b), the extracts are very active if MIC values <100 μg/mL, significantly active if 100 ≤ MIC ≤512 μg/mL, moderately active if 512 ≤ MIC ≤2048 μg/mL, and not very active if MIC >2048 μg/mL.

As of 6 November 2023, 487 cases of invasive *Streptococcus pyogenes* infection were reported to the Argentine National Health Surveillance System (SNVS 2.0) throughout the country. Of the people with invasive *Streptococcus pyogenes* infection, 78 were deceased. Almost half of the cases of *S. pyogenes* occurred in people who were under 16 years of age (241 cases, equivalent to 49.5%), while 38.5% of the fatal cases corresponded to children younger than 16 years of age, with the remaining cases distributed in all age groups (Ministerio de Salud Argentina., 2023). The advance of the *S. pyogenes* bacteria has not only occurred in Argentina. On 8 December 2022, the World Health Organization (2022) reported that at least five Member States in the European region (France, Ireland, the Netherlands, Sweden, and the United Kingdom of Great Britain and Northern Ireland) had reported an increase in the number of cases of the disease. *S. pyogenes* can cause mild infections and very serious and fatal illnesses. It can cause pharyngitis, which, when accompanied by a skin rash, becomes “scarlet fever.” It can also lead to pneumonia, infections located in the muscle, bone, or joints (known as fasciitis, osteomyelitis, and arthritis), and infections disseminated in the blood (WHO, 2022).

The UlDCM-s and Sephadex fractions F5–F7 were very active against nosocomial isolated *Streptococcus pyogenes* MQ4 with MIC values between 25 μg/mL and 50 μg/mL. The usnic acid showed a moderated activity against *S. pyogenes* MQ4 with a MIC/MBC equal = 25 μg/m, as well as toward MSSA, MRSA, and MRSA Q1 with MIC values in the range of 12.5–25 μg/mL. The yeasts *C. albicans* and *C. tropicalis* were inhibited by usnic acid, showing MIC/MBC values of 25/50 μg/mL. Various ranges or parameters for the evaluation of compounds and extracts with antimicrobial potential have been reported in recent decades. For compounds, the antibacterial activity parameters were significant (MIC ≤10 μg/mL), moderate (10 μg/mL < MIC ≤100 μg/mL), and low or negligible (MIC >100 μg/mL) (Kuete, 2010). In a previous report, isodivaricatic acid and divaricatinic acid showed antifungal effect toward *Microsporum gypseum* with a MIC of 50 μg/mL and against *Trichophyton mentagrophytes* and *T. rubrum* and with MIC values of 50 μg/mL and 100 μg/mL, respectively (Schmeda-Hirschmann et al., 2008). Usnic acid also showed moderate activity against *T. mentagrophytes* and *T. rubrum* with MIC values of 100 μg/mL and 200 μg/mL, respectively. Antimicrobial experiments with quantities higher than 1,000 μg/mL for extracts or 100 μg/mL for isolated compounds should be avoided, whereas the presence of activity is very interesting in the case of concentrations below 100 μg/mL for extracts and 10 μg/mL for isolated compounds (Ríos and Recio, 2005).

### 3.4 Nematicidal activity: *in vitro* mortality assay

*Meloidogyne* spp., which causes root-knot diseases in plants, is the most economically important plant-parasitic nematode worldwide (Ntalli and Caboni, 2012). The chemical nematicidal options have become more limited due to a large number of restrictions on their use. In this context, biopesticides and, specifically, bionematicides of botanical origin constitute a desirable component of pest management technology and practices (Ntalli and Caboni, 2012). Literature about lichens suggests that these have many antimicrobial, antifungal, insecticidal, and molluscicidal bioactivities (Silva et al., 2019; Agrawal et al., 2020). Ahad et al. (1991) assayed *Evernia prunastri* (belonging to the family *Parmeliaceae*) against J2 of *Toxocara canis* and identified the principal components. They found nematicidal activity and concluded that activity was attributed to compounds derived from 2,4-dihydroxybenzoate. Kim et al. (2018) evaluated the nematicidal activity of the non-lichenic fungus *Xylaria grammica* KCTC 13121BP against the J2 of *M. incognita* and their inhibitory effects against egg-hatching. To the best of our knowledge, there are no reports on the potential nematicidal activity of Argentinian lichens against *M. incognita*. The *in vitro* activity of *U. lethariiformis* extracts was assayed against J2 *M. incognita*, and the results are shown in Table 4. At 24 h, the *U. lethariiformis* extracts showed different levels of nematostatic effect toward J2. The UlDCM-i evidenced a higher nematostatic activity; however, it was not statistically similar to the effect produced by abamectin (positive control). Around 48 h, the effects of the extracts increased, and UlMeOH proved to be as active as UlDCM-i. At this time, they did not present significant differences with abamectin. At the end of the test at 72 h, the UlMeOH exhibited a total paralysis of the J2, followed by UlDCM-i. The mortality of the individuals at 72 h was confirmed, and the efficiency of the treatments (ET) was calculated according to the Schneider–Orelli formula. The registered mortality levels were, according to Faria et al. (2021), total for UlMeOH (100% ± 0.00), high for UlDCM-i (97.98% ± 2.77), and low for UlDCM-s (30.30% ± 15.73). The effect of the best treatments did not present a significant difference with the commercial nematicide (98.99% ± 2.26) (Table 4). Lower concentrations of UlMeOH, UlDCM-i, and abamectin were tested to establish the LC_50_ at 72 h (Table 5).

**TABLE 4:** Percentage of immobile J2 treated with Ul extracts at 0.5 mg/mL at 24 h, 48 h, and 72 h; and efficiency of the treatments (ET) at 72 h.

Treatments	% J2 immobile									ET (%)		
	24 h			48 h			72 h					
Negative control	0.00a	±	0	1.00a	±	2.24	1.00a	±	2.24	-	-	-
Positive control	95.00d	±	5	98.00c	±	2.74	99.00c	±	2.24	98.99b	±	2.26
UlMeOH	20.00b	±	5.77	96.25c	±	4.79	100.0c	±	0	100b	±	0
UlDCM-s	9.00a	±	4.18	36.67b	±	18.9	35.00b	±	14.7	30.3a	±	15.73
UlDCM-i	56.00c	±	13.4	97.00c	±	2.75	98.00c	±	2.75	97.98b	±	2.77

Different letters between columns indicate a significant difference (*p* < 0.05), means according to Fisher’s LSD test.

**TABLE 5:** LC_50_ and confidence interval (I.L: inferior limit and S.L: superior limit) to the UlDCM-i and UlMeOH extracts and abamectin (positive control).

Treatment	LC_50_ (µg/mL)	Confidence interval	
		I.L	S.L
UlDCM-i	320.76	280.71	360.46
UlMeOH	50.27	30.05	60.41
Abamectin	42.99	38.63	55.77

Molnár and Farkas (2010) suggested that lichens metabolize bioactive compounds that could be good candidates for formulating pesticides based on natural products, with the consequent lower environmental impact. Regarding usnic acid, one of the characteristic secondary metabolites of lichens of the genus *Usnea*, numerous authors have shown that it has activity against fungi and bacteria, comparable to the effect of streptomycin (Molnár and Farkas, 2010; Correché et al., 2004). Cetin et al. (2008) found that usnic acid has dose-dependent larvicidal activity against the third and fourth stages of *Culex pipiens*.

As previously mentioned, nature and its biodiversity provide numerous sources of agents with different bioactivities, including nematicidal activity. Recently, Manrique et al. (2023) evaluated the potential nematicidal properties of decoction (ZpDe), orange-yellow resin (ZpRe), and essential oil (ZpEO) from the Argentinean medicinal plant *Zuccagnia punctata.* The ZpDe, ZpRe, and ZpEO displayed strong nematicidal activity with LC_50_ values of 0.208 mg/mL, 0.017 mg/mL, and 0.142 mg/mL, respectively.

This is the first report on the potential nematicidal activity of Argentinian lichens against *M. incognita*.

## 4 Conclusion

The chemical profile and bioactive properties of extracts and fractions from the lichen *U. lethariiformis* were extensively studied. Seventeen compounds were detected, and of them, thirteen were identified through UHPLC-ESI-QTOF-MS analysis, including depsides, depsidones, fatty acids, dibenzofurans, benzoic acids, and triterpenes. The extracts and fractions exhibited diverse biological activities depending on the target. Regarding *T. cruzi*, the most active extract was UlDCM-s and its fraction 5. On the other hand, against *M. incognita*, the most active extracts were UlDCM-i and UlMeOH. Concerning bacteria, the methicillin-resistant *S. aureus* strain was most susceptible to the UlDCM-i extract, while the UlDCM-s extract was the most active against the yeast *C. albicans*. Our results indicated that *U. lethariiformis* may constitute a potential source of diverse bioactivities with application in the food, pharmaceutical, and agronomic industries.

## Data Availability

The original contributions presented in the study are included in the article/Supplementary Material, further inquiries can be directed to the corresponding authors.
