# Supplementary material for: Biological activities of Usnea lethariiformis lichen extracts and UHPLC-ESI-QTOF-MS analysis of their secondary metabolites
Source: Front Pharmacol. 2025 Jan 6;15:1508835. doi: 10.3389/fphar.2024.1508835 (PMC11742939; doi:10.3389/fphar.2024.1508835)

**Supplementary material**

**Figure S1.** HPLC DAD chromatogram (ThermoDionex 3000 RS) of crude methanolic extract of *U. lethariiformis* (UlMeOH).


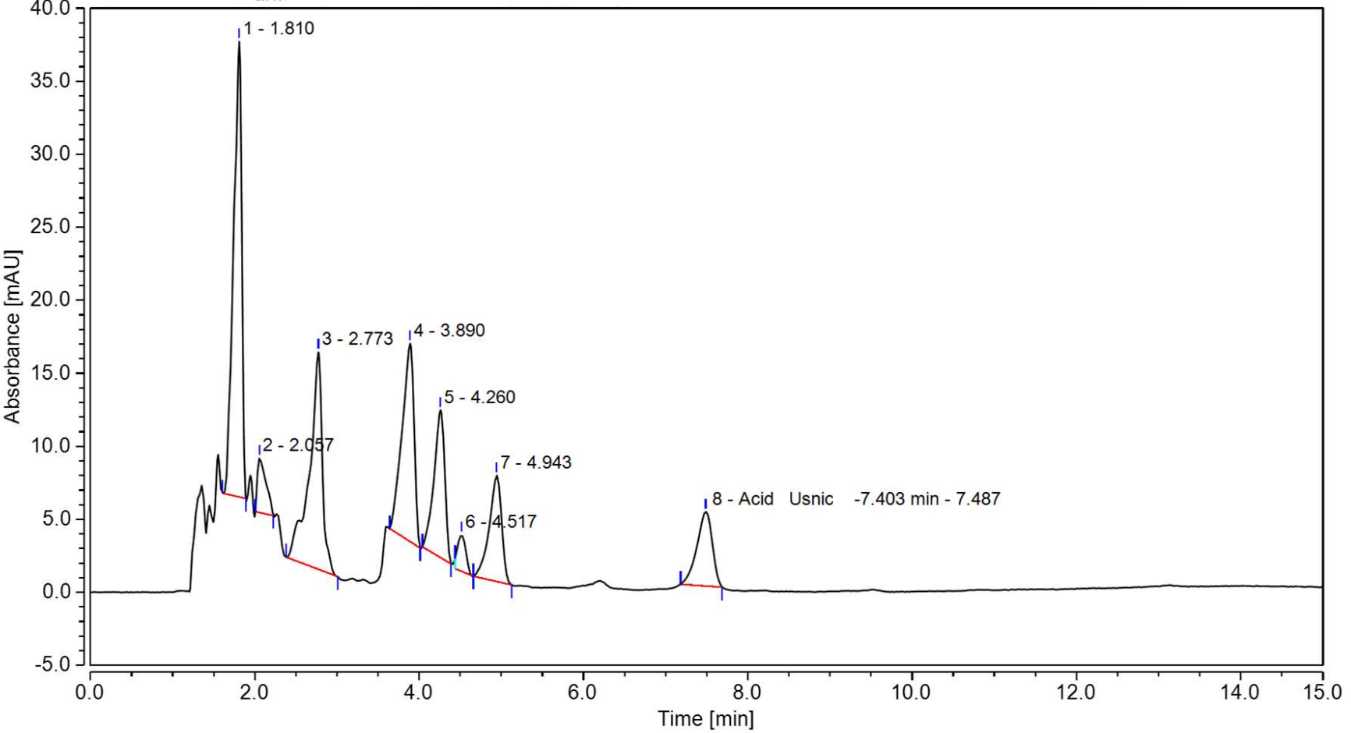


**Figure S2.** Chromatogram at 280 nm of Pure Standard of usnic acid and its DAD spectra


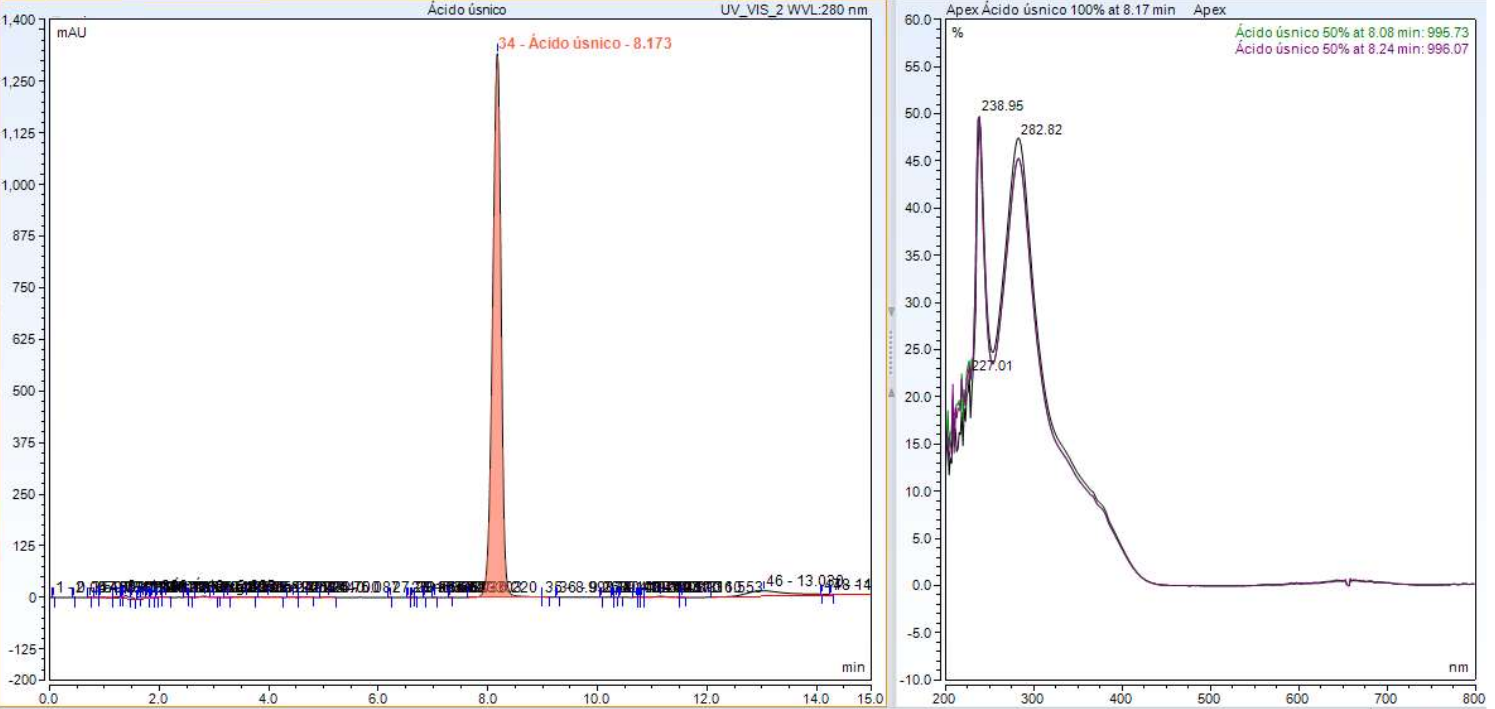


**Figure S3:** MS and MSn spectra of lichenic compounds detected by HR-MS in extracts of *U. lethariiformis*, (extracted using Bruker data analisis 4.0). Peak 1, connorstictic acid, Peak 2, stictic acid, Peak 3, divaricatinic acid, Peaks 4, diffractaic acid, Peak 5, squamatic acid, Peak 6, 4-O-demethyldivaricatic acid, Peak 7, dihydroxyheptadecatrienoic acid, Peak 8, divaricatic acid, Peak 10, barbatic acid, Peak 11, sekikaic acid, Peak 12, lichesterylic acid, Peak 13, barbatic acid, Peak 14, usnic acid, Peak 17, ursolic acid.


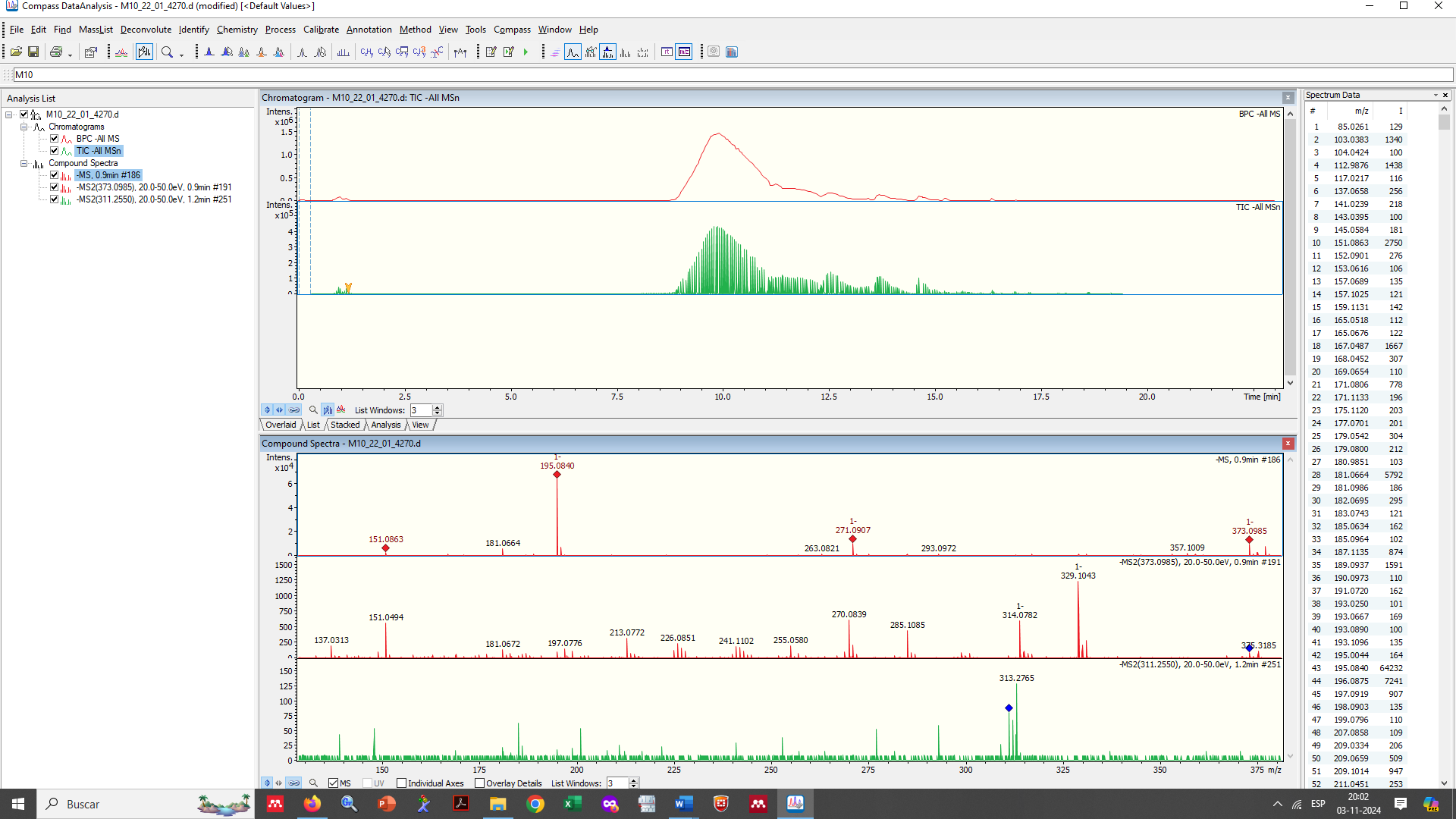

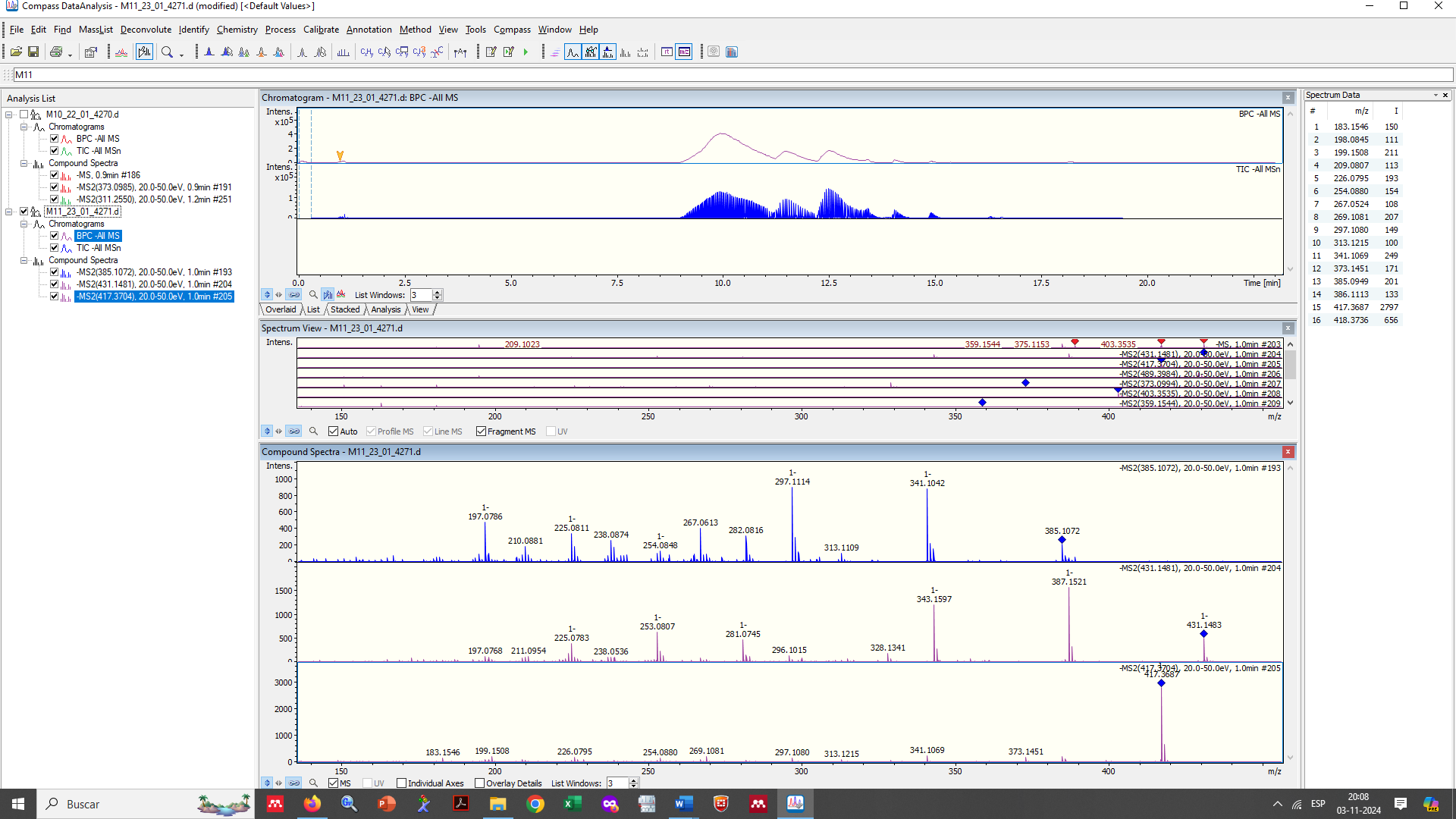


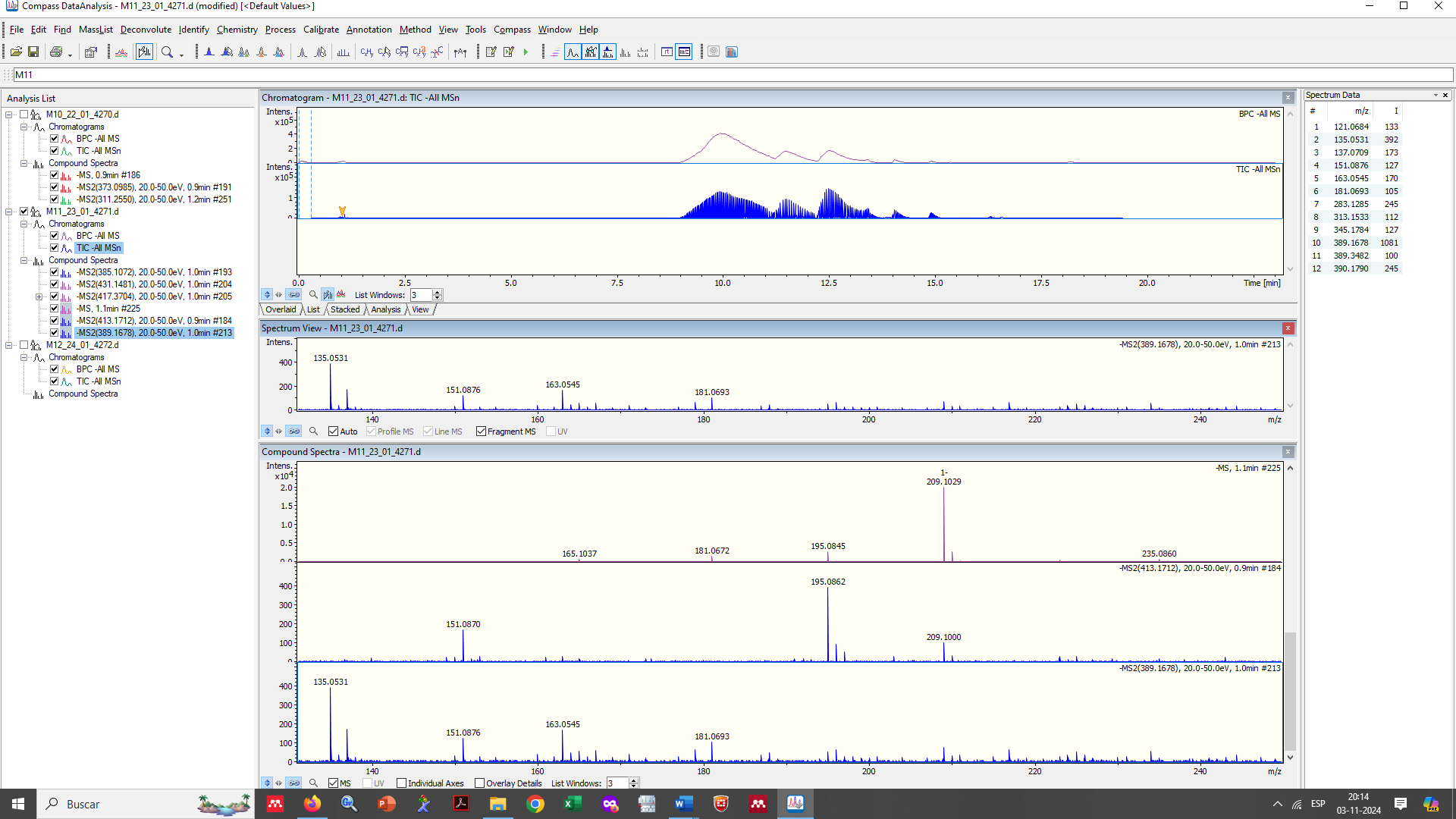

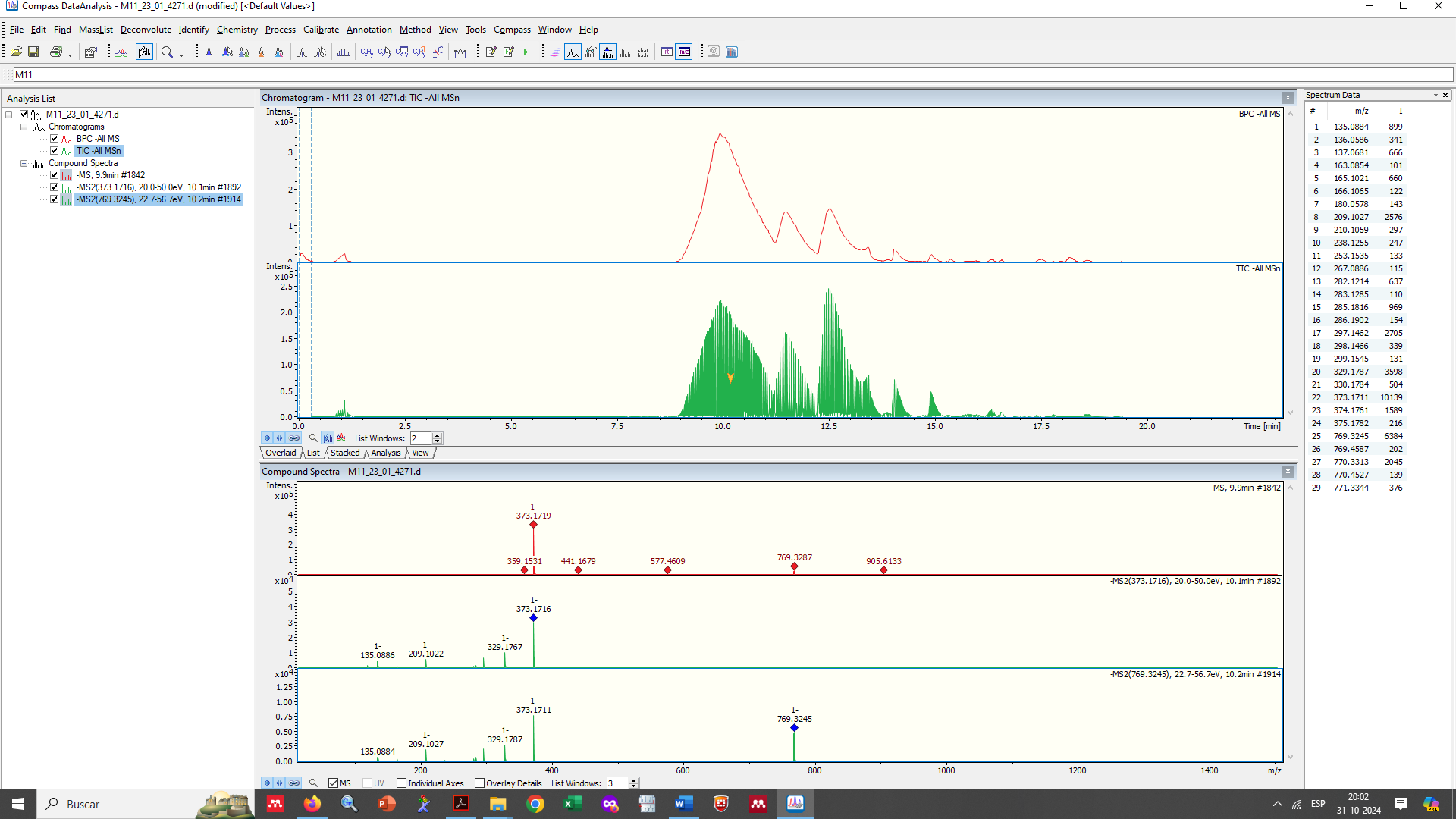


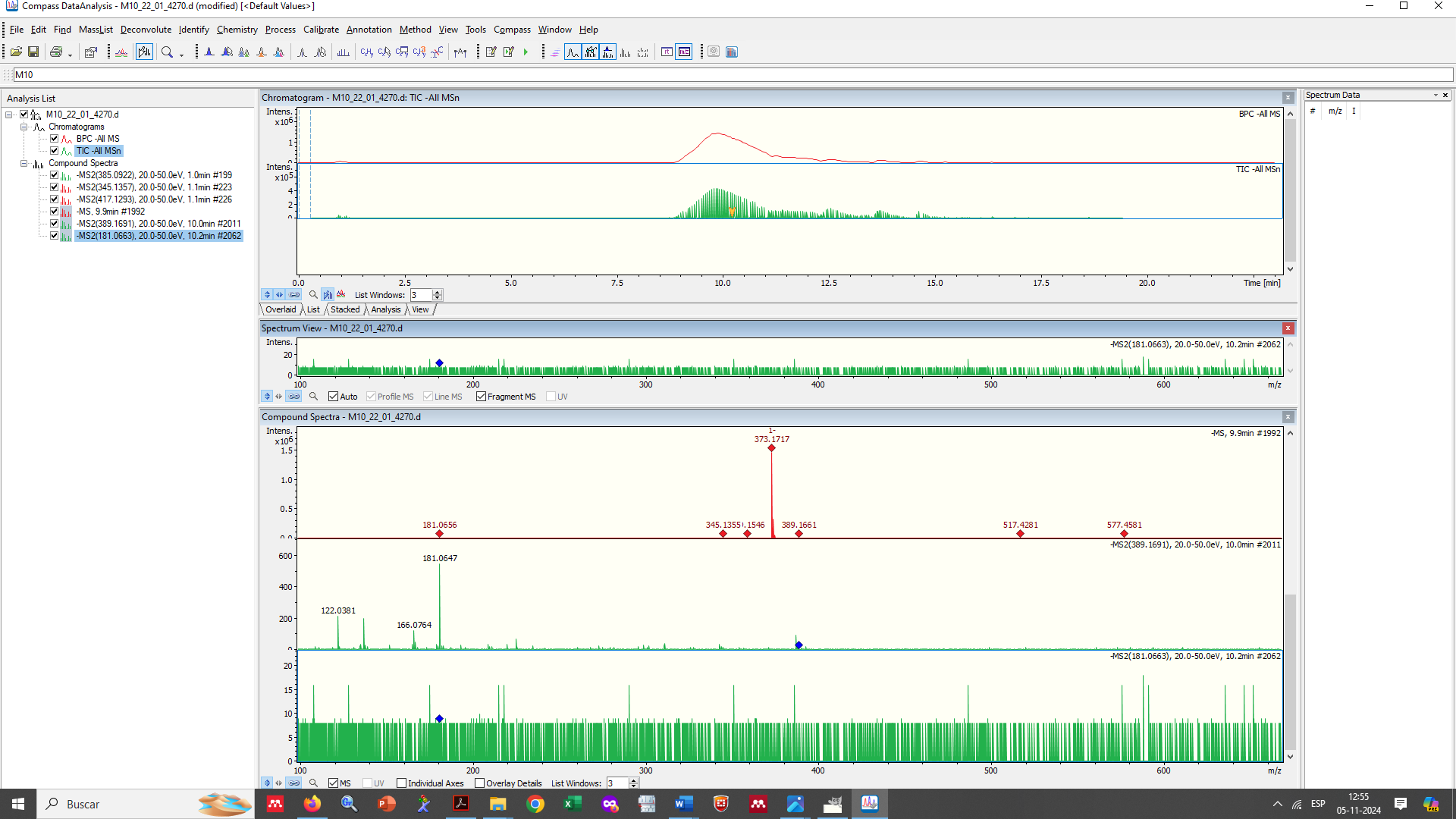

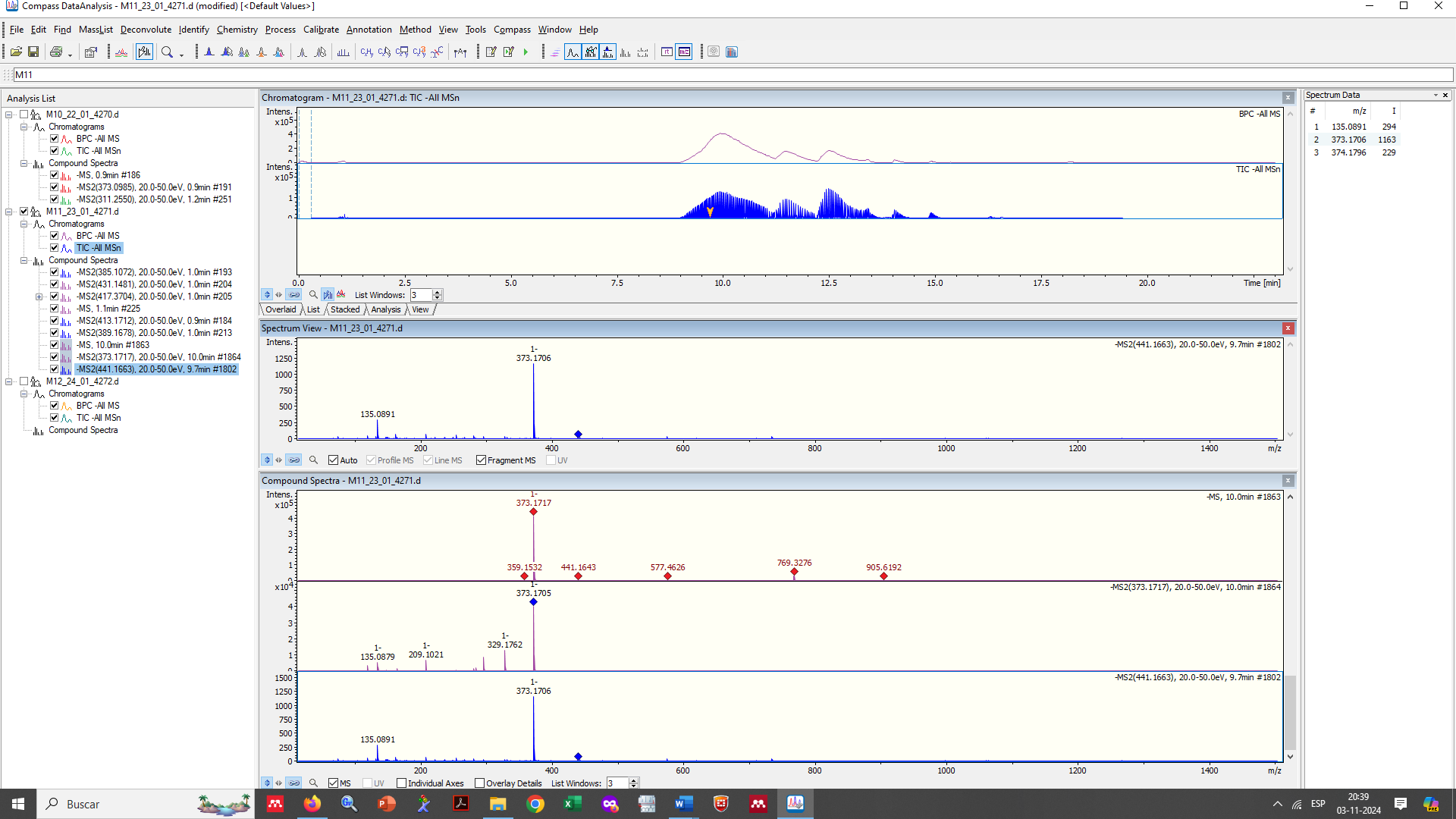


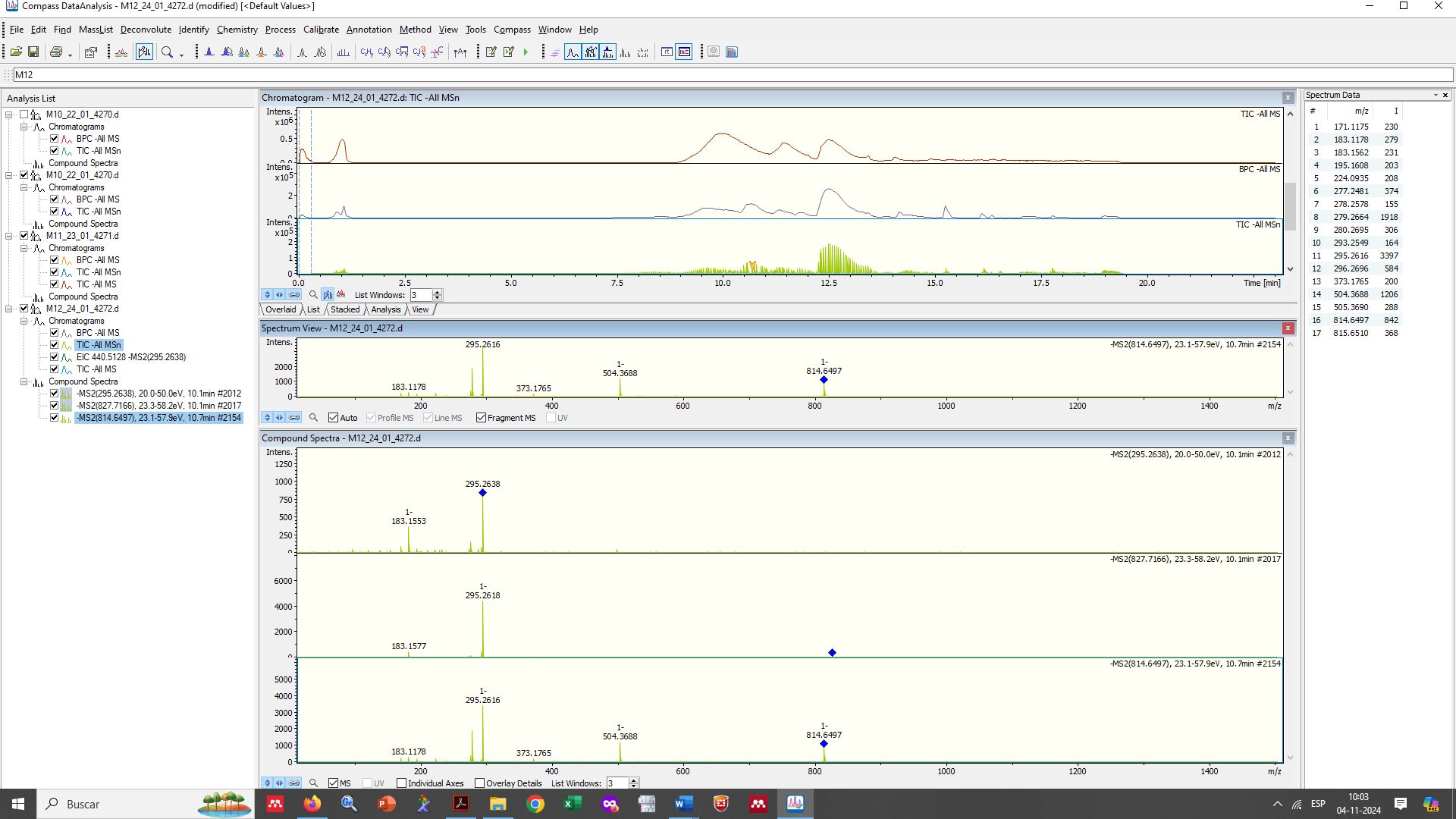

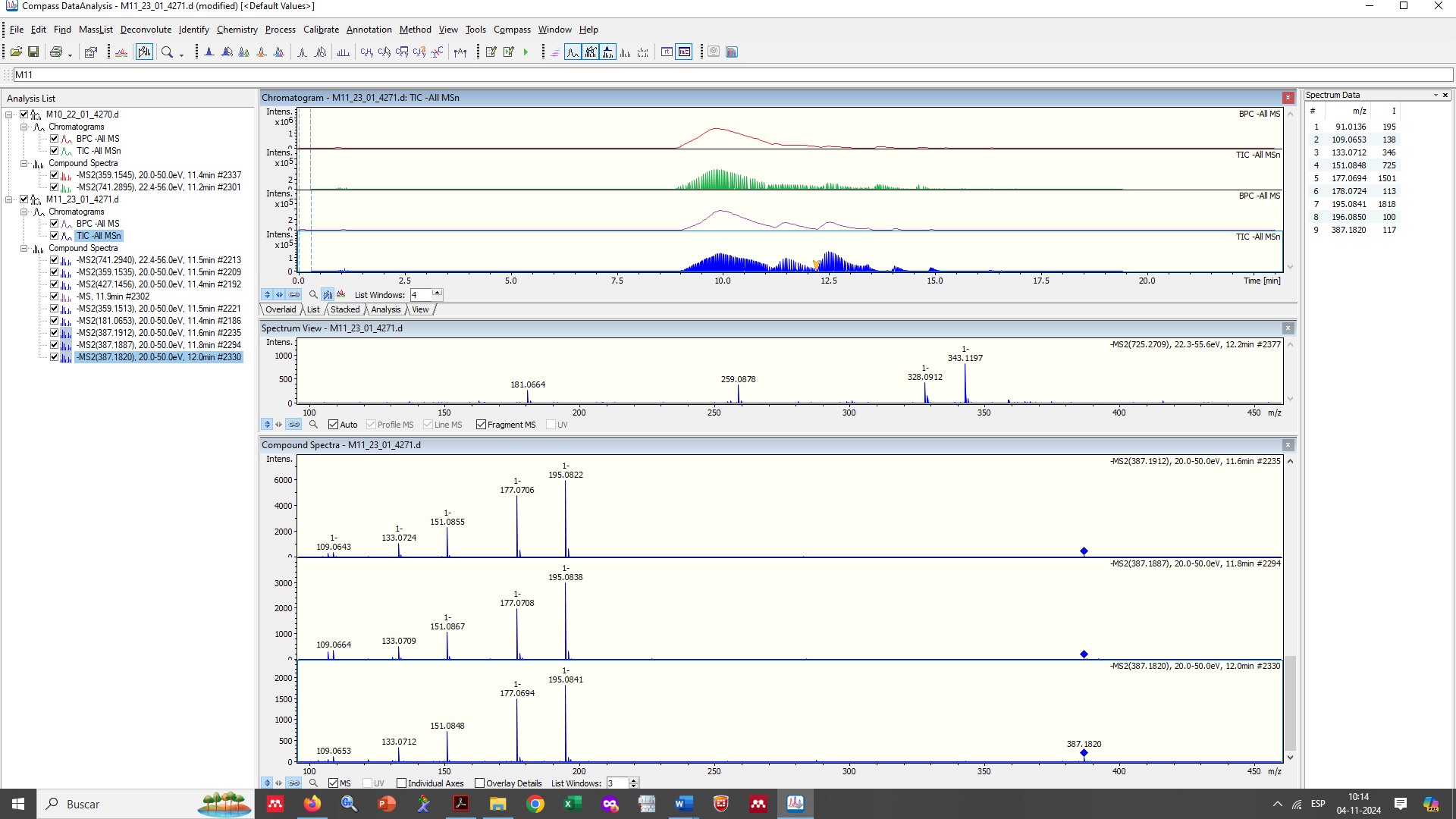


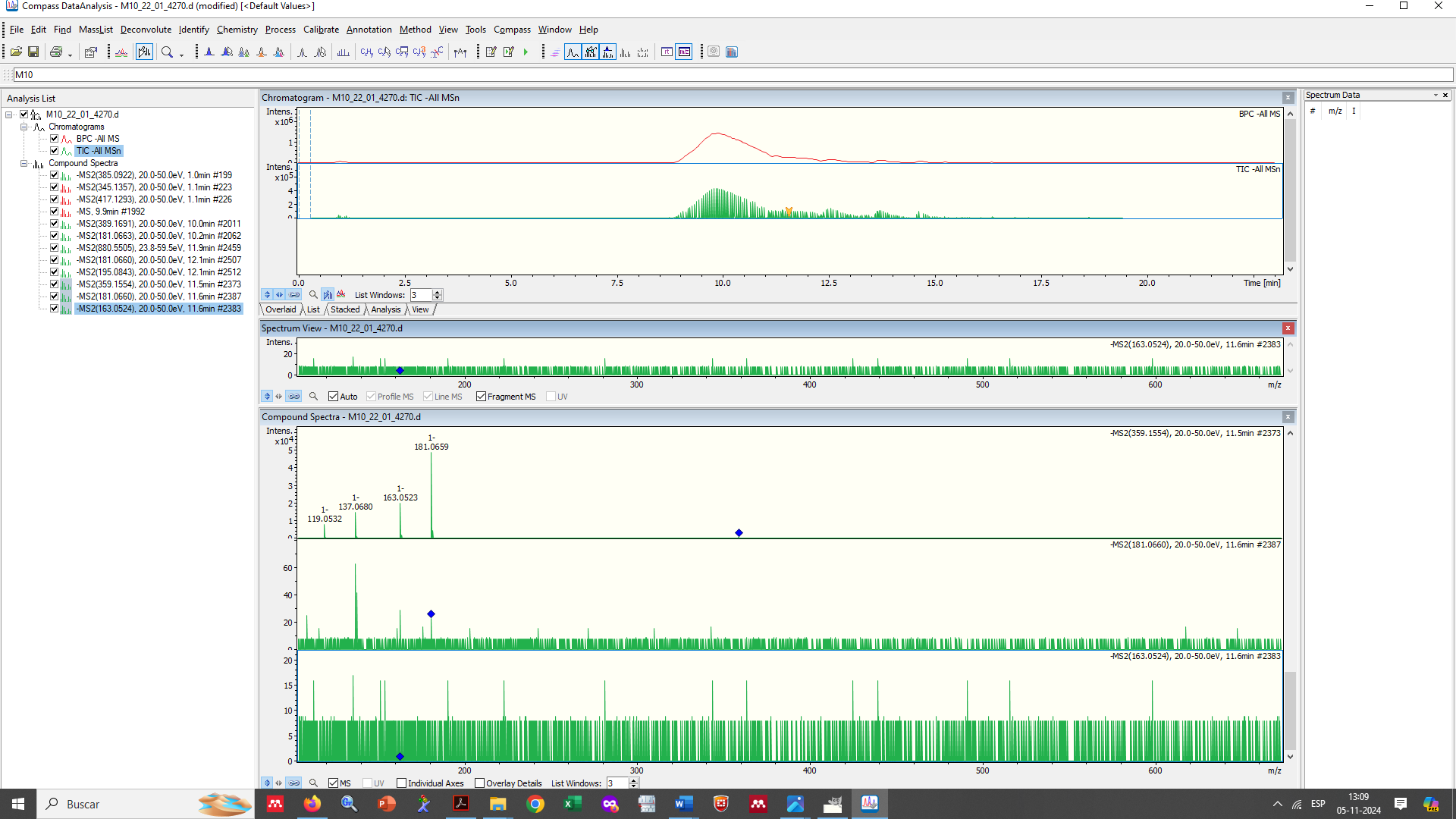

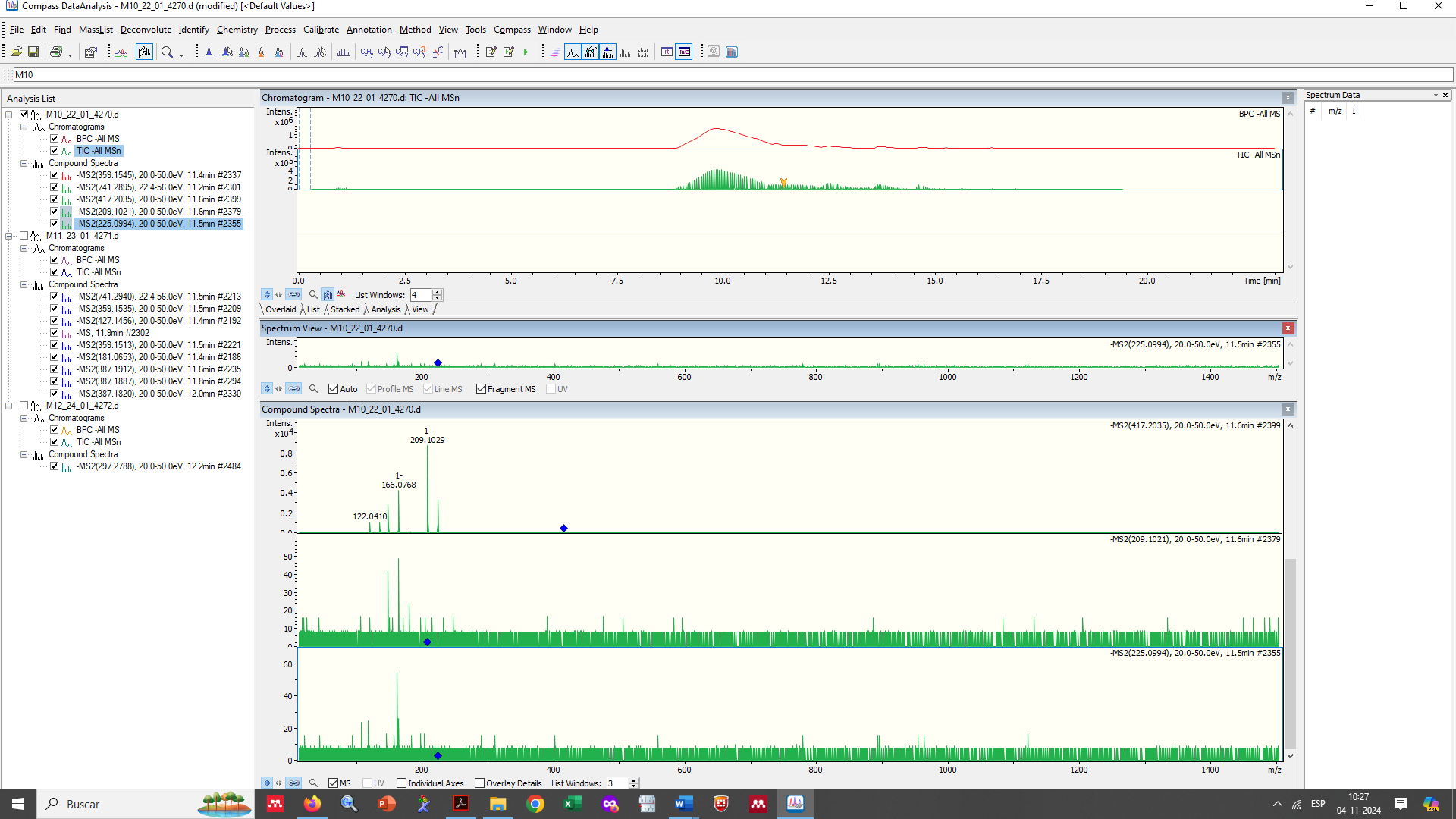

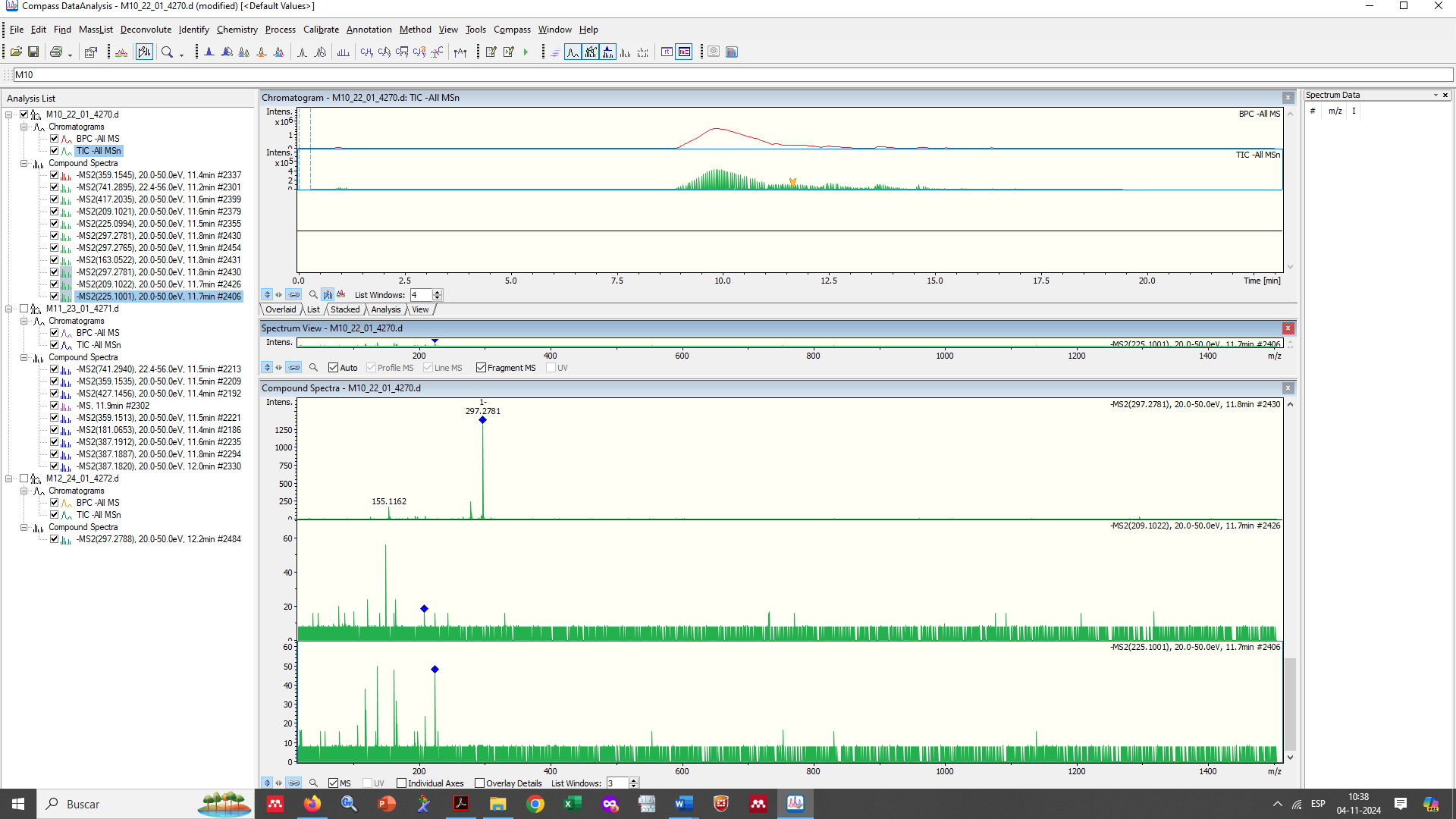

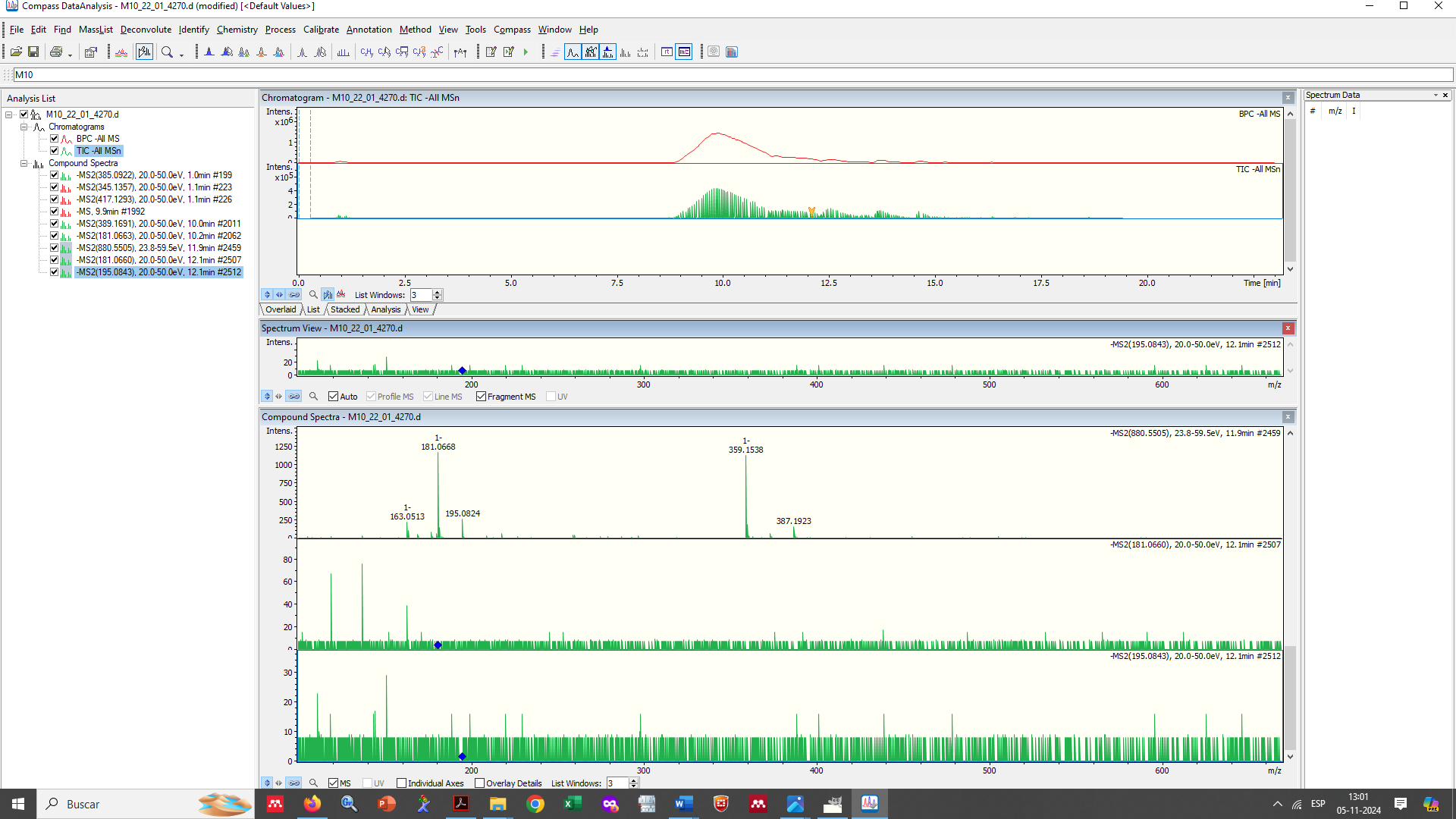

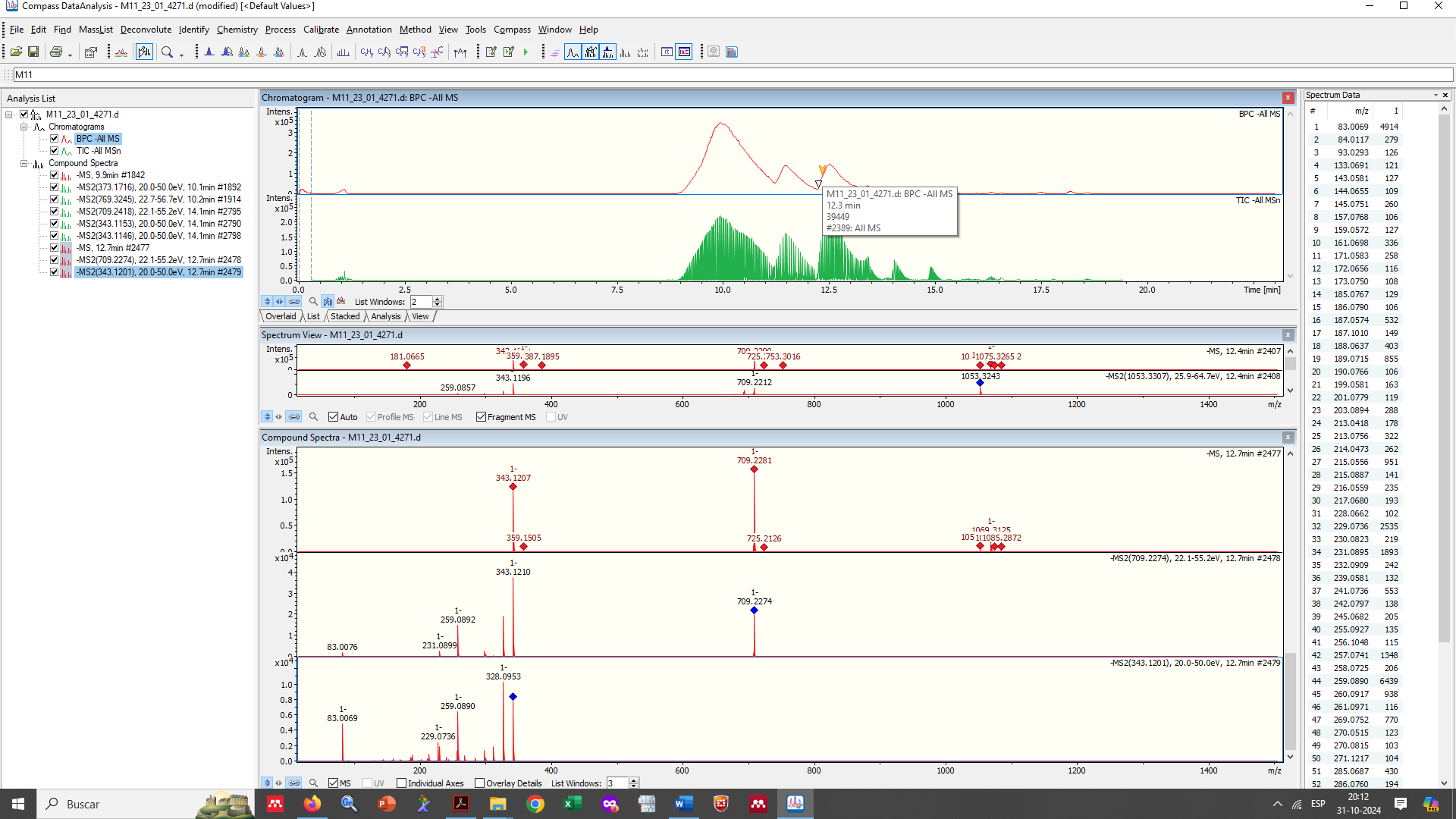

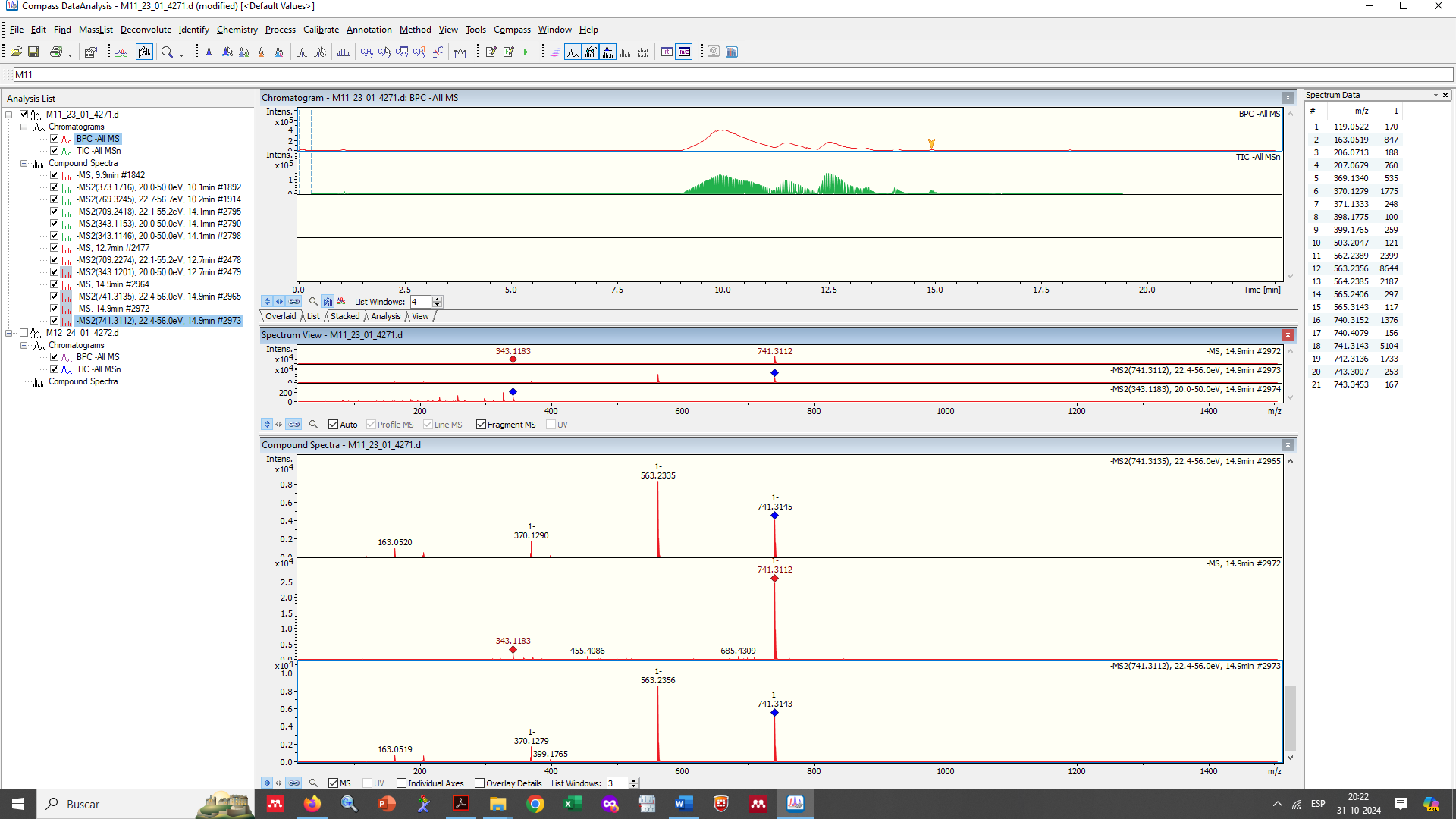


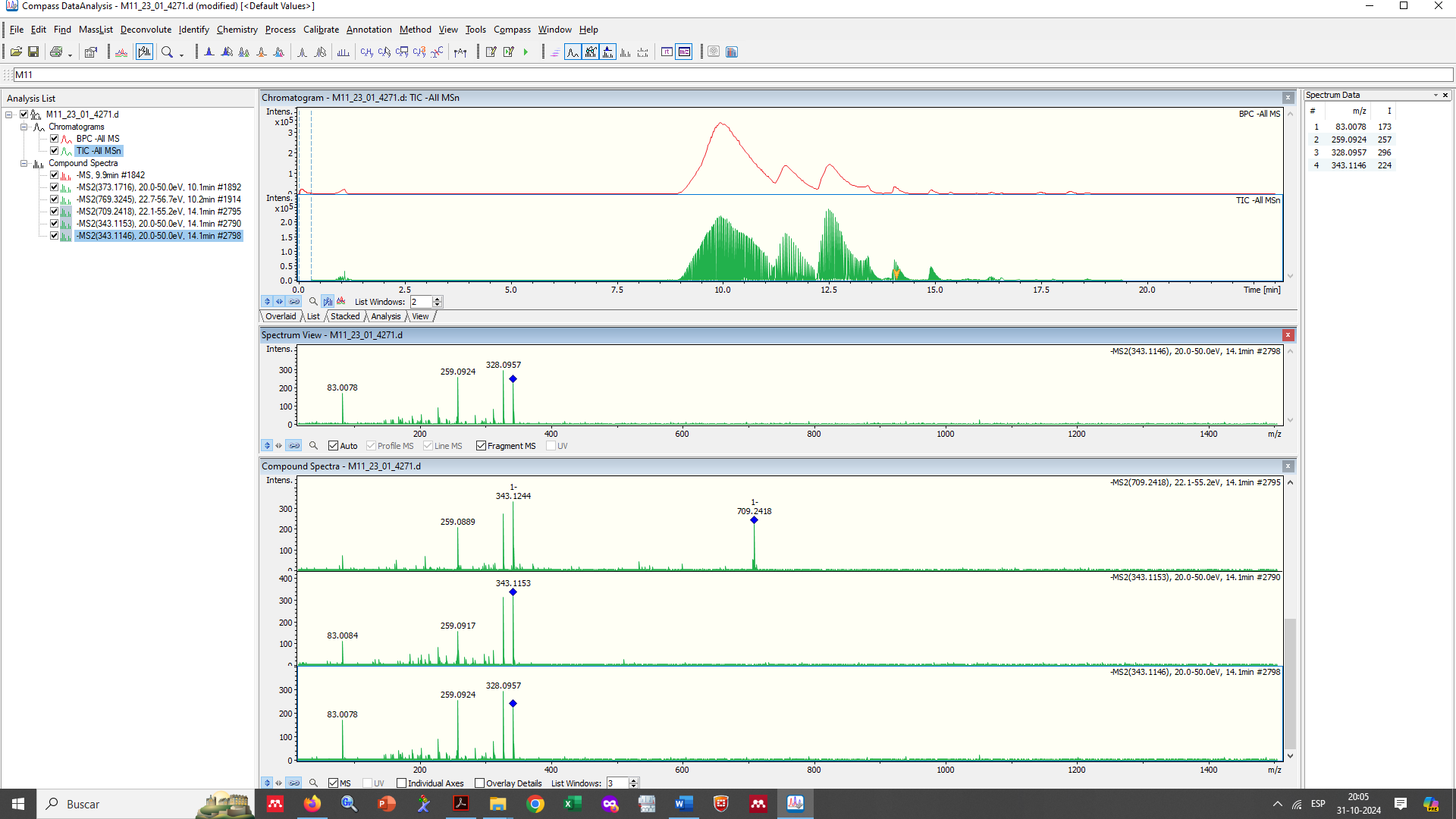

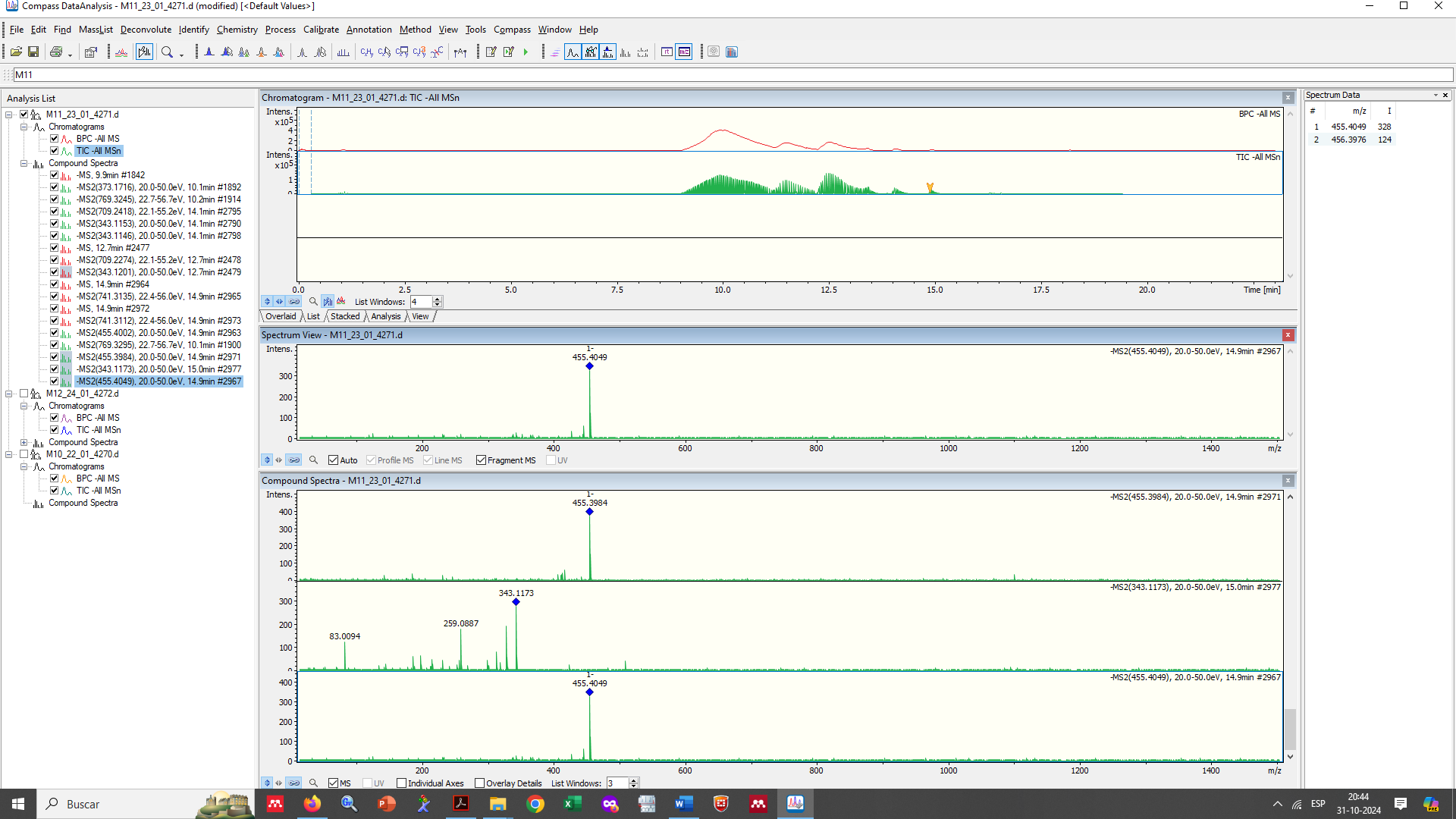

Supplement: Supplementary file 1 [file DataSheet1.docx]
